# Co-designing and pilot testing an infographic to support patients/families through the REMAP-CAP consent process: a mixed-methods study protocol

**DOI:** 10.1186/s40814-023-01290-6

**Published:** 2023-04-13

**Authors:** Heather K. O’Grady, Zahra Bhimani, Sandra Dalziel, Barbara Dolanjski, Gyan Sandhu, Marlene Santos, Kathy Smith, Srinivas Murthy, John C. Marshall, Michelle E. Kho

**Affiliations:** 1 Faculty of Health Sciences, School of Rehabilitation Sciences, McMaster University, Hamilton, Ontario Canada; 2 St. Michael’s Hospital Unity Health Toronto, Toronto, Ontario Canada; 3 Faculty of Medicine, Department of Pediatrics, University of British Columbia, Vancouver, Canada; 4 Physiotherapy Department, St. Joseph’s Healthcare, Hamilton, Ontario Canada

**Keywords:** Study protocol, Exploratory sequential mixed-methods research, Co-design, Study within a trial, SWAT, Informed consent, Consent interventions

## Abstract

**Background:**

Informed consent is critical to the ethical conduct of clinical research and requires understanding of a trial including its purpose, process, potential risks and benefits, and alternatives to participation. This can be challenging for complex trials, such as platform trials, and in high-stress environments, such as the intensive care unit (ICU). REMAP-CAP (randomized, embedded, multifactorial, adaptive platform trial for community-acquired pneumonia) is a platform trial which studies treatments for ICU patients with community-acquired pneumonia, including COVID-19. Patient/family partners (PFP) identified challenges during the REMAP-CAP consent process.

**Methods:**

This is a patient-centred co-design study to refine and test an infographic to supplement current REMAP-CAP consent documents. Infographic prototypes were developed by patients, substitute decision-makers (SDMs), and researchers with lived experience in the ICU or with ICU research. We will apply a two-phase exploratory sequential, mixed-methods research design. In phase 1, we will conduct focus groups with ICU patients, SDMs, and research coordinators (RCs). We will use inductive content analysis to inform infographic refinement, to be pilot tested in phase 2. Phase 2 is a prospective study within a trial (SWAT) at ≤ 5 REMAP-CAP sites. We will collect self-reported data from patients/SDMs and RCs. The primary outcome is feasibility (eligible consent encounters, receipt of infographic, consent to follow-up, completion of follow-up surveys). Data will be integrated to understand if/how quantitative results build upon the qualitatively informed infographic.

**Discussion:**

Phase 1 results will be used to co-design an infographic, directly informed by the perspectives of patients, SDMs, and RCs involved in ICU research consent discussions. Results from phase 2 will determine the feasibility of infographic implementation in REMAP-CAP consent encounters. These feasibility data will inform a larger SWAT to evaluate our consent infographic. If successful, use of a co-designed infographic to support REMAP-CAP consent documents may improve the experience of consent for patients, SDMs, and RCs.

**Trial registration:**

The Northern Ireland Hub for Trials Methodology Research SWAT Repository (SWAT no. 176)

**Supplementary Information:**

The online version contains supplementary material available at 10.1186/s40814-023-01290-6.

## Background

Informed consent is essential to the conduct of ethical clinical research and requires clear communication and understanding of a trial’s purpose, methods, potential risks and benefits, and alternatives to participation [1, 2]. This can be challenging for platform trials (also called adaptive trials or multi-arm, multistage designs), which simultaneously evaluate multiple treatments for a disease [3, 4]. Platform trials allow researchers to identify superior treatments and test new treatments as they emerge; thus, the treatments offered are constantly changing [3]. The complexities of a platform trial can be difficult to understand, particularly for patients and families approached for consent.

Ideally, a consent discussion occurs in a calm, non-stressful environment with potential participants in a positive mindset, with ample time to review consent documents before making a decision [2]. This is not always possible when research involves vulnerable populations, such as patients in the intensive care unit (ICU). Patients requiring ICU care have a life-threatening illness, creating stress for patients and families. Traditional consent forms are long and scientific, requiring significant time and energy to explain and understand, making them challenging to use in stressful, time-constrained situations [5, 6].

Platform trials have been crucial in the timely identification of optimal treatment interventions for individuals with COVID-19 in the ICU [7, 8]; one of these is REMAP-CAP (randomized, embedded, multifactorial, adaptive platform trial for community-acquired pneumonia) [9]. REMAP-CAP is an international trial with 36 Canadian sites. This trial adds complexity to the traditional two-arm randomized trial consent through multiple randomizations with different risk-benefit profiles, response-adaptive randomization (where better performing arm(s) are preferentially randomized), and international coordination and data sharing.

### The problem

In Canada, REMAP-CAP is led by a team of researchers and clinicians who developed the Canadian Adaptive Platform Trial in Intensive Care (CAPTIC) research program [10]. CAPTIC is a Canadian Institutes of Health Research (CIHR)-Strategy for Patient-Oriented Research (SPOR)-funded research program. The CAPTIC research program formed the CAPTIC Patient/Family Partners (PFP), including patients and families with lived ICU experience, to gain their perspectives and uphold the program’s commitment to conducting patient-oriented and guided research. The PFP’s first priority was to address challenges during ICU research consent to improve communication between researchers, patients, and families.

PFP reported the consent documents for the REMAP-CAP trial were difficult to understand given the trial complexity. In addition, patients and families are highly vulnerable when they are approached for research consent. Patients emergently requiring ICU care typically have a life-threatening illness, creating high stress and anxiety [5, 11]. Often, patients are unable to provide first-hand consent due to the severity of their illness, and consent is sought from a patient’s substitute decision-maker (SDM) [5]. A SDM is in an individual, typically a patient’s family member or other legal representative, who makes decisions on a patient’s behalf when they are unable [5]. The eligibility timeframe for enrollment in ICU-based clinical trials can be very short, adding further strain to the consent encounter [5]. Given the unique challenges of research consent in the ICU, clear communication with patients and SDMs is necessary to allow them the opportunity to choose to participate in clinical research. PFP believe use of an infographic may aid in this communication. It is essential that those with lived experience are involved in the development of such a tool, which can be facilitated through co-design [12, 13].

### Co-design approach

We will adopt human-centered design (HCD) approach to reimagine the REMAP-CAP consent process with those central to the experience [patients, SDMs, research coordinators (RCs)] [14, 15]. HCD consists of four consecutive phases: discover, define/ideate, prototype, and test/redesign. HCD embraces the active involvement and established role of the CAPTIC PFP. Our co-design team includes ten diverse individuals [three PFP, Rehabilitation Science PhD Candidate, REMAP-CAP program manager (responsible for coordinating patient and SDM engagement), three REMAP-CAP investigators, two REMAP-CAP RCs]. Our study will focus on the prototype and rest/redesign phases.

### Objectives

The overall objective of this mixed-methods study is to co-design and pilot test an infographic to augment the standard REMAP-CAP consent process. The objective of phase 1 is to understand patient, SDM, and RC perspectives on infographic prototypes. The objective of phase 2 is to determine feasibility of infographic implementation in REMAP-CAP consent encounters. The use of mixed methods will allow us to develop an infographic that is relevant to patients, SDMs, and RCs though in-depth qualitative data collection while also using quantitative data to measure the feasibility of using the infographic.

## Methods

We used four guidelines to inform protocol reporting:

- 1) The Mixed-Methods Article Reporting Standards (MMARS) [16]
- 2) The Journal Article Reporting Standards for Qualitative Research (JARS-Qual) [16]
- 3) The Standard Protocol Items: Recommendations for Interventional Trials (SPIRIT) statement and recommendations for pilot and feasibility trials [17, 18]
- 4) The guideline for reporting health design research [19] (reporting checklists in Additional file 1).

### Overview

This will be conducted as a study within a trial (SWAT) embedded in the REMAP-CAP trial (NCT02735707) [9, 20]. This SWAT will be jointly coordinated at St. Michael’s Hospital Unity Health Toronto (Toronto, Ontario) and McMaster University (Hamilton, Ontario).

**Fig. 1:**
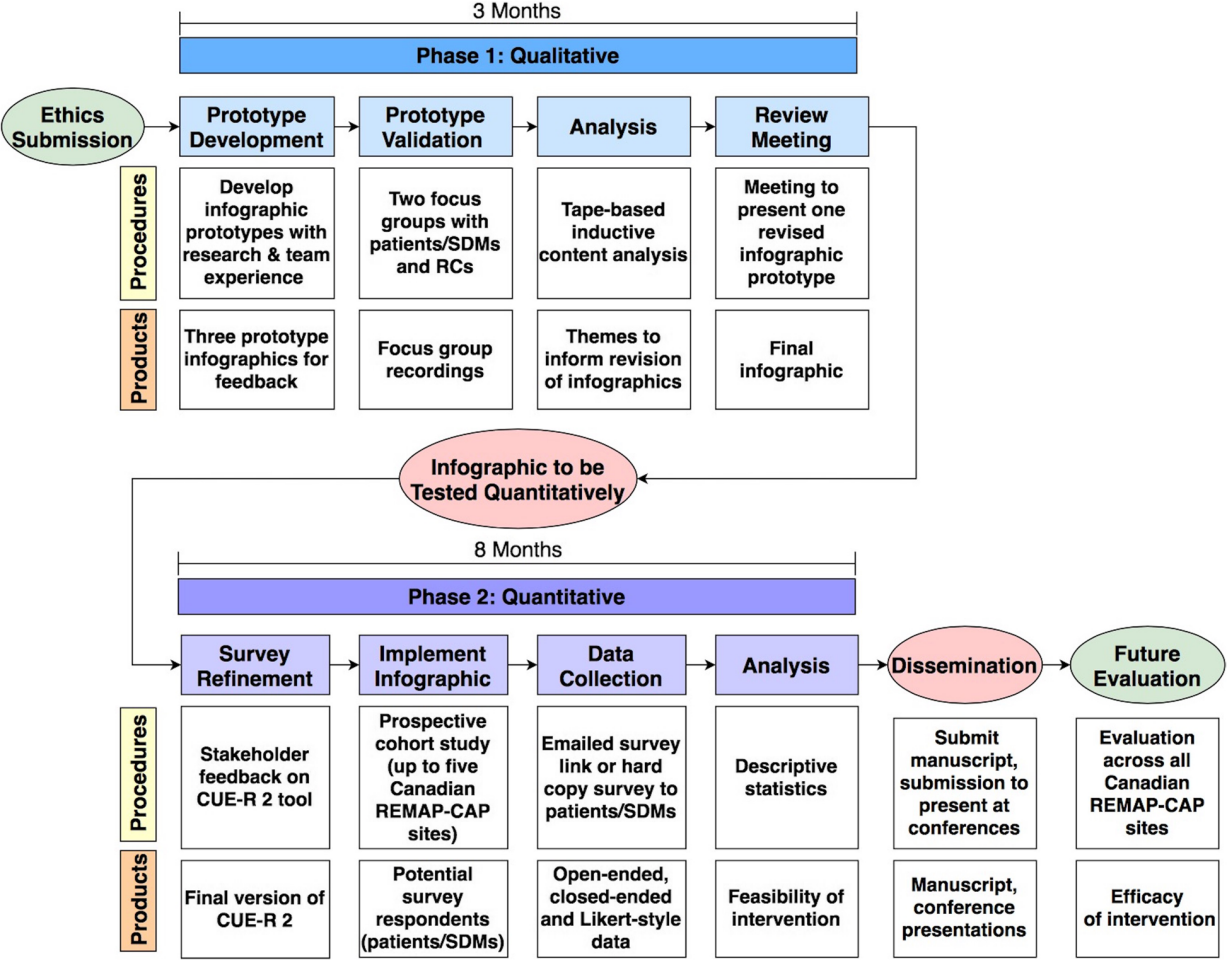
Study flow diagram. This diagram presents an overview of study procedures and products. Blue boxes represent the first qualitative phase (infographic development), while purple boxes represent the second quantitative phase (pilot and feasibility testing. Red circles represent points of mixed-methods data integration. SDMs, substitute decision-makers; RCs, research coordinators; CUE-R 2, Consent Understanding Evaluation-Revised 2; RCT, randomized controlled trial

We will apply an exploratory sequential, mixed-methods research design (Fig. 1) [21]. This design is characterized by two phases: (1) qualitative data collection and analyses to inform infographic refinement and (2) testing the infographic through quantitative data collection and analyses [21]. Integration will occur at two points: first, the results of phase 1 will be used to refine the infographic which will be pilot tested in phase 2, and second, the results of phase 2 will be used to understand if and how they expand upon results from phase 1.

### Research ethics approval

This study has been approved by the Unity Health Toronto Research Ethics Board (ID no. 3779).

#### Phase 1 methods: infographic refinement

### Design

Phase 1 involves two semi-structured focus groups, a stakeholder review meeting, and will follow the principles of qualitative description. We will explore participant perspectives of infographic prototypes, with analysis remaining close to the data [22].

### Setting

Participants will be recruited from within Canada. All activities will occur remotely via Zoom, an externally hosted cloud-based videoconferencing service (San Jose, CA, USA: Zoom Video Communications Inc.). We will also use a web-based visual collaboration platform, Miro (Miro, San Francisco, CA, USA), to facilitate discussion during focus groups.

Eligibility criteria are as follows:

• Adults ≥ 18 years
• Patients *or* SDMs with lived experience in the ICU *and* RCs with experience consenting patients or SDMs for REMAP-CAP
• Able to read, write, and speak in English
• Access to Internet and technology (e.g., computer, tablet, mobile phone) to participate in video calling *or* access to a telephone for those who may choose not to participate in video calling

### Outcomes

The primary outcome is an in-depth understanding of patient, SDM, and RC perspectives of infographic prototypes through key themes from focus groups, to inform infographic refinement.

### Sampling, recruitment, and consent

We will use two sampling strategies to identify potential participants: purposive criterion sampling, where participants are intentionally selected based on predefined eligibility criteria, and snowball sampling, where participants recommend other individuals for participation [23, 24]. We will recruit participants from two distinct stakeholder groups, patients, SDMs, and RCs.

Patients and SDMs have the critical role of making an informed decision to consent to participation and enrolling themselves/their loved one in a clinical trial in the ICU. We will recruit a diverse group of patients and SDMs approached for consent in any ICU research study. We will use four key strategies to invite patients and SDMs to join our study: (1) CAPTIC Patient Panel — individuals who have previously expressed interest in patient-engagement activities related to CAPTIC and REMAP-CAP; (2) Twitter; (3) REMAP-CAP site study update meetings; and (4) Critical Care/Patient Engagement Networks. Efforts to build relationships with community-based research organizations, social service agencies, and healthcare organizations are ongoing. Typically, these groups have their own volunteer, patient committees, and distribution lists, such as the SPOR Support Units, the Health Quality Ontario’s Patient, Family and Public Advisor Network, the South Asian Health Research Hub, Access Alliance, and the Yee Hong Geriatric Care Centre. In our recruitment materials and communications, we will explicitly invite individuals from underrepresented populations and those not commonly represented in research to join our study. Recruitment materials will direct interested individuals to the REMAP-CAP website where they will complete a short questionnaire; a member of the study team will then follow-up and confirm eligibility.

RCs play an important role in the informed consent process, from the design and development of consent documents, to approaching individuals for participation in the study. Thus, our second stakeholder group includes RCs who seek consent from patients or SDMs in the ICU to participate in REMAP-CAP. We will recruit RCs using two strategies: (1) REMAP-CAP site study update meetings, and (2) we will email current REMAP-CAP RCs.

Efforts to accommodate varied schedules and availabilities, while compensating for time and expertise, are also being made, to ensure panelists can participate, regardless of their occupation and socioeconomic status. Participation in the focus group will be considered implied consent. We will use member checking throughout focus groups to ensure accuracy in interpretation of responses, by verbally asking participants whether our interpretation is correct [25]. With participants’ consent, focus groups will be audio recorded using Zoom’s built-in recording software. Participant timeline and activities are summarized in Table 2.

### Sample size

We will recruit 4–6 participants from each of our two stakeholder groups, each with 3–4 patients/SDMs and 2 RCs, for a total of 8–12 participants [26].

### Data collection

We will host two semi-structured focus groups for feedback on infographic prototypes and use two methods of data collection: focus-group transcripts and field notes. Based on preliminary research and conversations with the study team, we have developed three infographic prototypes (Additional File 2) which will be presented to stakeholders. Prototype 1 was developed by our research team; however, after receipt of funding and further discussion, a decision was made to consult a design expert who developed prototypes 2 and 3. This design consultant is external to the REMAP-CAP team and is trained in co-design and HCD methodology and will facilitate the focus groups and analyze data. Given that the design consultant developed prototypes 2 and 3, we will engage an additional co-facilitator, who did not develop prototypes, to increase transparency in the process of infographic revision.

Both focus groups will cover the same content with the same questions and structure. We developed a preliminary semi-structured facilitation guide which will be pilot tested with members of the study team who were not involved in development prior to data collection (Additional File 3) [24]. The purpose of pilot testing is to ensure correct understanding and interpretation of questions and will inform revisions to the facilitation guide to improve clarity, as needed. Focus groups will be a maximum of 120 min in length, allowing detailed discussion of specific elements of the infographic prototypes, including flow, content, organization, imagery, relatability, appropriateness, and ease of understanding. Before the focus groups, participants will receive a pre-work document which will describe the purpose and objectives of the focus groups and guide participants through a preliminary review of the infographic prototypes. Pre-work materials will take approximately 1.5–2 h to complete and will be sent 2 weeks before the focus-group dates (Additional File 4).

Data collected during focus groups will inform refinement and development of one final infographic prototype. All participants will be invited to a stakeholder review meeting by Zoom where the revised infographic will be presented. The purpose of this meeting is to elicit any final feedback and to achieve consensus among study participants on a final infographic for pilot testing. Field notes will be documented after focus groups and the stakeholder review meeting to provide an audit trail of researcher experience and decisions [27].

### Data management

Focus-group and stakeholder review meeting audio recordings will be stored in Zoom’s Cloud service (Canada) and manually transcribed. All study documents (e.g., audio recordings, transcripts, field notes) will be password protected, stored on the McMaster University secure network, and only research team members will have access to these files. Audio recordings will be deleted immediately following completion of data analysis for both phases of this study. No personal identifying information will be requested. Any personal identifying information voluntarily given during the focus groups will be deleted from the final transcripts. The focus-group transcripts, as well as all contact information, and other research data will be stored securely on encrypted, password-protected servers at McMaster University.

### Data analysis

We will use a “tape-based” approach for analysis of focus-group data [26]. Tape-based analysis involves listening to focus-group-audio recordings during analysis versus analyzing transcripts in a textual format. We will use inductive content analysis [28]. This includes open coding (making notes and assigning codes), creating categories (grouping similar codes), and abstracting data (to represent each category) [29]. Categories identified during analysis will be grouped into themes which will serve as the results of phase 1 and will provide an in-depth understanding of participants’ perspectives and experiences, to inform infographic refinement.

### Confidentiality

We will take necessary precautions to ensure participant privacy and data safety (meeting passwords, use of domains for participants, locking meetings once started, allowing participants to change/abbreviate their name). While our local research ethics boards have approved the use of Zoom for data collection, there is a small risk of a privacy breach for data collected on external servers. We will offer participants the opportunity to make alternative arrangements (e.g., phone interview) if they have concerns. At the start of the focus groups and review meeting, we will review the purpose of the study, describe the role of participants, and inform participants that they may discontinue participation at any time.

### Remuneration

We will provide participants with a CAD $100 gift card to compensate for their time. This is intended to recognize participants’ time and important contributions; however, we do not believe this is enough to coerce study participation. Compensation will not be revoked upon focus-group cessation.

#### Phase 2 methods: pilot testing

### Design

This is a SWAT embedded in the REMAP-CAP trial. We will conduct a prospective cohort study at up to five Canadian REMAP-CAP sites.

### Setting

Phase 2 will be initiated at St. Michael’s Hospital Unity Health Toronto in Toronto, Ontario, Canada. We selected this as the primary site as it is the Canadian REMAP-CAP Regional Coordinating Center. St. Michael’s Hospital Unity Health Toronto has a 29-bed mixed medical-surgical ICU. After initiation of the pilot, we will scale up to include up to four additional REMAP-CAP sites (maximum five sites) in Southern Ontario. These sites will be selected based on willingness to participate and capacity to implement the intervention.

Eligibility criteria for SWAT consent encounters are as follows:

• Patients *or* SDMs approached to participate in REMAP-CAP
• RCs conducting consent encounters using the infographic
• Patients or SDMs able to read, write, and speak in English
• Patients or SDMs able to receive REMAP-CAP consent documents either in person or by email

Exclusion criteria are as follows:

• Patients enrolled in REMAP-CAP by deferred consent

### Recruitment and consent

#### RCs

We will contact REMAP-CAP site RCs by email and invite them to participate in this SWAT. RCs who consent to participate in this SWAT will screen patients for eligibility for the REMAP-CAP trial as usual and include the infographic as part of their standard verbal description of the REMAP-CAP trial. At the conclusion of each consent encounter, regardless of their decision to participate, the RC will inform the patient/SDM that our research team is working to improve the consent experience for future trial participants and invite them to participate in a follow-up survey.

#### Patient/SDM

REMAP-CAP requires written consent for study participation. All participants will receive the standard REMAP-CAP consent documents with a verbal description of the trial. Eligible patients, or their SDMs, who are approached for participation in REMAP-CAP with the infographic will automatically be eligible for this SWAT. At the conclusion of the REMAP-CAP consent discussion, patients or SDMs will be invited to participate in a follow-up survey. If the patient/SDM agrees to follow-up and can receive and complete an electronic survey, the RC will advise the patient/SDM that a member of the research team will send an email within 24 h to seek feedback on their consent experience. The RC will inform the patient/SDM that the decision to provide feedback will not impact quality of care and the RCs will not see their responses. The RC will provide the SWAT research team with the participant’s name and contact information by email or by phone. The follow-up email, sent centrally by the SWAT research team, will outline survey objectives, invite the individual to participate, and will contain an electronic survey link.

For patients/SDMs who are unable to receive or complete an electronic survey, we will provide the option to complete a paper version. If the patient/SDM agrees to follow-up, the RC will provide them with a paper copy in an unsealed envelope. These patients/SDMs will be asked to complete the paper survey within 1 week and return it to the ICU research office. Site RCs will be asked to scan and email completed surveys to the SWAT research team for central data entry.

At the beginning of the survey, we will inform potential respondents that their participation is voluntary, that completed questionnaires will remain confidential, and provide contact information where a member of the team can address any questions. For electronic questionnaires, we will send nonrespondents a reminder email 1 week after the initial email. Before recontacting patients and/or SDMs, the SWAT research team will communicate with site RCs to ensure it is an appropriate time for communication. Participant timeline and activities are summarized in Table 1.

**Table 1:**
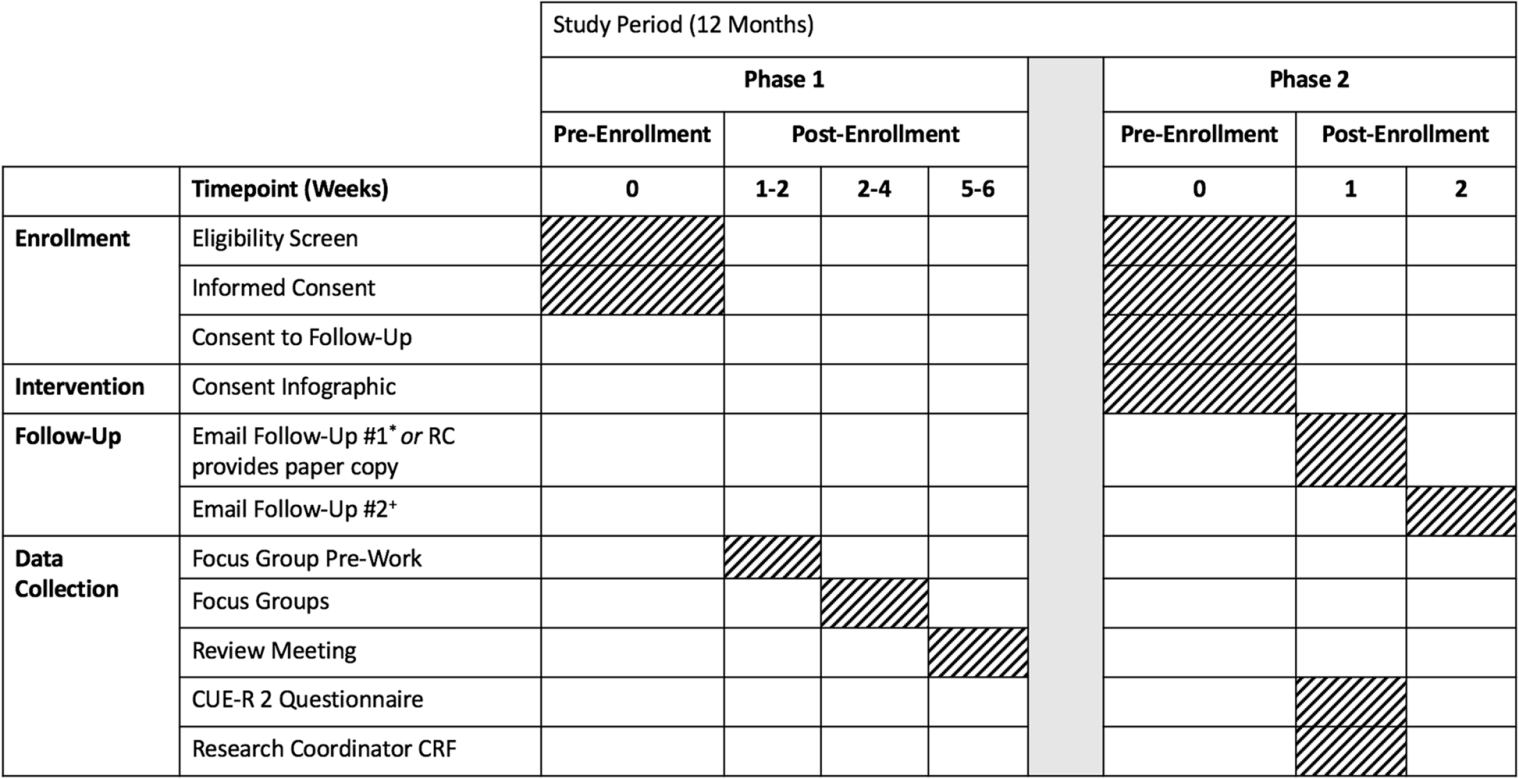
Standard Protocol Items: Recommendations for Interventional Trials (SPIRIT) figure

Legend: This table summarizes participant activities for both phases of this study, which will take place over a 12-month period. *The first email follow-up will be sent to all patients/SDMs who consent to follow-up and will contain an electronic link to the CUE-R 2 questionnaire. +The second email follow-up will be sent to patients/SDMs who do not complete the CUE-R 2 1 week after the initial email was sent. Abbreviations: *CUE-R 2*, Consent Understanding Evaluation-Revised 2 Tool; *CRF*, case report form

### Sample size

We will use a sample of consecutive REMAP-CAP consent encounters at each of our study sites. Due to funding and study personnel constraints, we will recruit patients/SDMs for up to 6 months. Based on historical recruitment rates, we anticipate 2 patients per month, per site, for a total of 60 patients/SDMs. We conducted a sample size calculation using a confidence interval approach and expect our target sample size of 60 patients/SDMs to be sufficient to achieve our feasibility objectives [30]. We selected eligible consent encounters as our most important outcome, thus basing our calculation on a proportion of ≥ 68%, alpha = 0.1 and a margin of error of 10%, for a sample size of 60. We will seek feedback from all RCs consenting during this time. We anticipate 1–2 RCs per site for a total of 5–10 RCs. We will meet with RCs at each site before study implementation.

### Intervention

We will provide patients/SDMs with the infographic from phase 1 to augment the standard REMAP-CAP consent process. The standard consent process includes consent documents provided to the patient/SDM and an explanation/discussion of the study between the patient/SDM and study RCs. For consent encounters that occur in person, a paper copy of the infographic will be provided to the patient/SDM with the standard consent documents. For remote consent encounters (by telephone or videoconference), an electronic copy of the infographic will be provided with the standard consent documents.

### Outcomes

The primary outcome of this pilot study is feasibility. For patients/SDMs, this will include receipt of the infographic, consent to follow-up, and the completion of follow-up surveys. For RCs, this will include successful implementation of the infographic. We will collect additional data regarding the feasibility and acceptability of this intervention using patient-centered outcomes. We will collect data from both patients/SDMs and RCs who participate in the consent encounters.

#### Patients/SDMs

We will use a modified version of the Consent Understanding Evaluation-Revised tool (CUE-R) [31]. The CUE-R is a structured interview tool which was developed through a literature review and expert opinion, and validated through focus groups with research participants and community advisory board members, and later expanded to assess satisfaction with the consent process and consent documents [31, 32]. We modified the CUE-R to a self-administered survey, include both patients and SDMs as potential survey respondents, and fewer questions. Modifications were made through consensus among our multidisciplinary SWAT research team, including patients/SDMs, REMAP-CAP RCs, and investigators. We called our modified version of the CUE-R tool the “CUE-R 2” (Table 2). Participants who choose to complete an electronic survey will receive a unique survey link through LimeSurvey that will prevent duplicate responses. Both electronic and paper versions of the CUE-R 2 will conclude with five demographic questions including gender, age, race, highest level of education, and previous participation in medical research.

**Table 2:** Phase 2 outcome measures, constructs, and questions

		**Question type (no. of questions)**		
	**Outcome**	**Likert style**	**Close-ended**	**Open-ended**
**CUE-R 2 (26 questions)**				
** Feasibility**	Consent mode & decision		2	
	Extent infographic was used	1		
	Ease of use of infographic	2		
	Acceptability of infographic	1		3
** Effectiveness**	Trial-related knowledge		4	
	Extent standard consent documents were used	1		
	Ease of use of consent documents	2		
	Satisfaction with information received	1	2	
	Desire for further information	1		
	Confidence in consent decision	1		
	Demographics		5	
**Research coordinator CRF (15 questions)**				
** Feasibility**	Consent mode & decision		3	
	Duration of consent encounter		1	
	Successful implementation of infographic		4	
	Ease of use of infographic	1		
** Effectiveness**	Satisfaction with consent encounter	1		
	Number & difficulty of questions asked	1	1	
	Perception of patient/SDM comprehension	1		
	Perception of patient/SDM satisfaction	1		
	Perception of patient/SDM confidence	1		

Legend: This table summarizes outcome measures for phase 2. The CUE-R 2 electronic survey will be completed by patients/SDMs who receive the consent infographic. The research coordinator (RC) CRF will be completed electronically by RCs who use the infographic during consent encounters with patients/SDMs. Abbreviations: *CUE-R 2*, Consent Understanding Evaluation-Revised 2 Tool; *CRF*, case report form

#### RCs

**Fig. 2:**
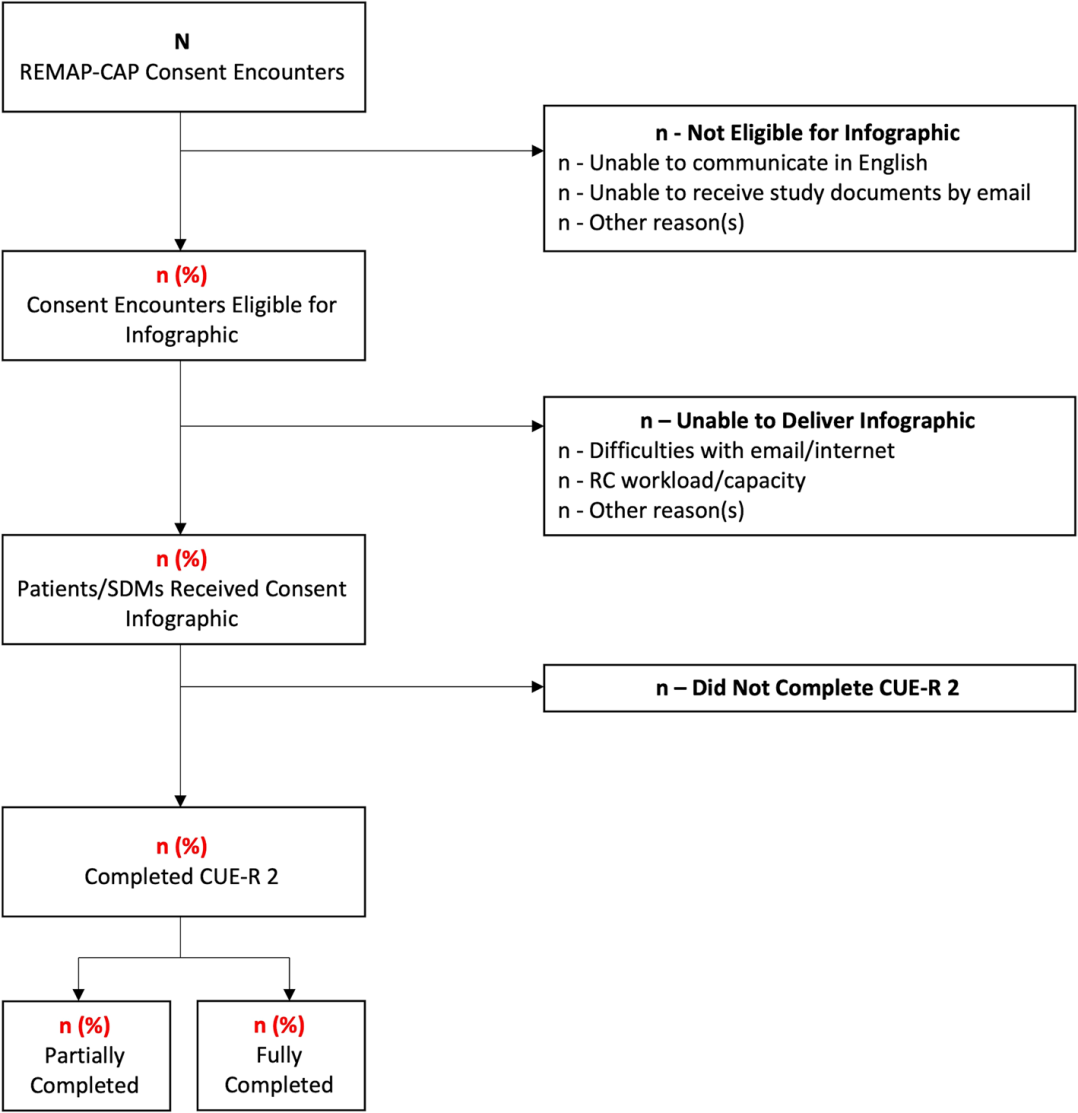
Flowchart of phase 2 study participants and feasibility outcomes. *n* represents number. Red text represents feasibility metrics. CUE-R 2, Consent Understanding Evaluation-Revised 2 Tool

We will collect data using a modified version of a case report form (CRF) used in a SWAT of video-augmented consent for an ICU rehabilitation trial [33]. Outcomes are summarized in Table 2 and Fig. 2. RCs will be asked to complete an electronic CRF on LimeSurvey for each consent encounter within 24 h, to decrease the potential influence of recall bias [34].

### Data management

Raw data and analysis files will be password protected and stored on a password-protected computer. We will keep a copy of data files on the McMaster University secure network, and only SWAT research team members will have access. Completed hard copy surveys will be stored in a locked filing cabinet in a locked office.

### Data analysis

Data will be analyzed using Stata (v. 15.0, StataCorp LP, College Station, TX, USA). For the CUE-R 2, we will analyze demographic and survey response data using descriptive statistics, including using counts, frequencies, and means (standard deviations) or medians (1st, 3rd quartiles) for Likert-style questions or if data are skewed. We will narratively summarize text data from open-ended questions. We will calculate survey response rate as the proportion of completed surveys compared to the number of patients/SDMs who are invited to participate [35]. We will also calculate the proportion of partially completed and fully completed surveys. We will analyze RC CRFs using descriptive statistics. We will assess success of implementation using three metrics: (1) eligible consent encounters (proportion of patients/SDMs identified to receive the infographic compared to total number of REMAP-CAP consent encounters during the study period ≥ 68%, based on potentially eligible consent encounters in an ICU-based SWAT of video consent [33]); (2) receipt of infographic (number of patients/SDMs who received the infographic as a proportion of eligible consent encounters, ≥ 80%, suggested as the lower limit for high intervention fidelity [36]); and (3) feasibility of data collection will be assessed by consent rate for follow-up [≥ 71%, based on a systematic review of consent rates for trials in the ICU (lower limit of 95% confidence interval for median consent rate) [37]] and the survey response rate (≥ 71, based on the survey response rate in an ICU-based SWAT of video consent [33]).

### Confidentiality

Emails will be stored separately from survey responses; a master log containing email information will be created for this purpose. All email communications will be through a generic study email, hosted on the McMaster University secure domain.

### Remuneration

After survey completion, we will provide survey respondents with a CAD $5 gift card. This is intended to recognize participants’ time and important contributions; however, we do not believe this is enough to coerce study participation. Compensation will not be revoked in the case of incomplete survey responses. We will also provide RCs at our pilot sites with a CAD $50 gift card in appreciation of their time and expertise in implementing our study protocol.

### Dissemination policy

We will disseminate results through a peer-reviewed publication, national and international presentations.

### Mixed-methods integration

Data integration will occur at two points in this study [21]. The first, primary point of integration is before phase 2, where qualitative focus-group data will be used to develop a consent infographic that will be tested quantitatively. Once phase 2 is complete, we will integrate the results from both phases to understand if and how the quantitative results build upon the qualitatively informed infographic. Transcripts from phase 1 will be used during this point of integration. This second point of integration will inform future evaluation of the infographic in a future randomized trial across REMAP-CAP sites. Lastly, we will use investigator triangulation (multiple researchers involved in data collection and analysis) and methodologic triangulation (multiple methods of data collection) to improve the validity of our results [38].

## Discussion

### Strengths and limitations

Our study is patient-led, patient-oriented, and patient-centric. We partnered with patients and SDMs with lived ICU research consent experience who ideated this project. They are members of our research team, and their engagement will ensure the relevance of our work for patients and SDMs. Our multi-site pilot study will identify feasibility challenges through detailed data collection and feedback from stakeholders. These data will be critical to future implementation and evaluation of the consent infographic.

Representation of the 36 heterogeneous Canadian REMAP-CAP sites and contexts was a challenge. We chose to pilot the infographic at five sites in Southern Ontario, because of their high historical recruitment rates for REMAP-CAP. We will pilot the infographic in English, limiting our representation of non-English-speaking patients and SDMs who utilize the Canadian healthcare system. However, in our future evaluation study, we will include additional sites (nationally and internationally) and will consider translation to additional languages.

### Significance

Feasibility data are essential to optimize implementation and evaluation of a new intervention. Results from this study will be used to scale up and evaluate our consent infographic at additional REMAP-CAP sites. If successful, an infographic to support the traditional consent model could be more broadly tested. Use of a co-designed infographic during the consent process may benefit all stakeholders. Patients and SDMs may better comprehend trial information and increase their knowledge of REMAP-CAP, while RCs may experience improved patient/SDM understanding. In combination, these improvements may benefit the study through increased RC satisfaction, increase patient/SDM satisfaction, and well-informed consent decisions.

## Supplementary Information

**Additional file 1.** 

**Additional file 2.** 

**Additional file 3.** 

**Additional file 4.**

## Data Availability

Not applicable

## Abbreviations

ICU: Intensive care unit
SDM: Substitute decision-maker
REMAP CAP: Randomized, embedded, multifactorial, adaptive platform trial for community-acquired pneumonia
CAPTIC: Canadian Adaptive Platform Trial in Intensive Care
PFP: CAPTIC patient/family partners
HCD: Human-centered design
RC: Research coordinator
SPIRIT: Standard Protocol Items: Recommendations for Interventional Trials
SWAT: Study within a trial
CUE-R: Consent Understanding Evaluation-Revised tool
CRF: Case report form
