# Supplementary material for: Co-designing and pilot testing an infographic to support patients/families through the REMAP-CAP consent process: a mixed-methods study protocol
Source: Pilot Feasibility Stud. 2023 Apr 13;9:58. doi: 10.1186/s40814-023-01290-6 (PMC10098229; doi:10.1186/s40814-023-01290-6)
Supplement: Supplementary file 1 — Additional file 1. [file 40814_2023_1290_MOESM1_ESM.docx]

**Additional File 1**

Table of Contents

Mixed Methods Article Reporting Standards (MMARS) checklist. 2

Journal Article Reporting Standards for Qualitative Research (JARS-Qual) checklist. 3

Standard Protocol Items: Recommendations for Interventional Trials (SPIRIT) checklist. 5

Guideline for reporting health research design checklist. 9

Phase one letter of information. 11

Phase two letter of information. 14

Stakeholder review meeting agenda and overview. 17

**Mixed Methods Article Reporting Standards (MMARS) checklist.**

| **Item** | **Page #** |
| --- | --- |
| **Title** |  |
| Refrain from using words that are either qualitative (e.g., "explore," "understand") or quantitative (e.g., "determinants," "correlates"), because mixed methods stands in the middle between qualitative and quantitative research. Reference the mixed methods, qualitative methods, and quantitative methods used. | 1 |
| **Abstract** |  |
| Indicate the mixed methods design, including types of participants or data sources, analytic strategy, main results/findings, and major implications/significance. | 2-3 |
| **Introduction** |  |
| State three types of research objectives/aims/goals: qualitative, quantitative, and mixed methods. Order these goals to reflect the type of mixed methods design used. | 6 |
| Describe the ways approaches to inquiry were combined, as it illuminates the objectives and mixed methods rationale | 8 |
| **Methods** |  |
| Explain why mixed methods research is appropriate as a methodology given the paper’s goals. | 6 |
| Identify the type of mixed methods design used and define it. | 7 |
| Indicate the qualitative approach to inquiry and the quantitative design used within the mixed methods design type | 7, 8, 13 |
| If multiple approaches to inquiry were combined, describe how this was done and provide a rationale (e.g., descriptive, interpretive, feminist, psychoanalytic, postpositivist, critical, postmodern, constructivist, or pragmatic approaches), as it is illuminating for the mixed method in use. | N/A (did not use multiple approaches) |
| Provide a rationale or justification for the need to collect both qualitative and quantitative data and the added value of integrating the results (findings) from the two databases. | 6, 7 |
| When data are collected from multiple sources, clearly identify the sources of qualitative and quantitative data (e.g., participants, text), their characteristics, and the relationship between the data sets, if there is one (e.g., an embedded design). | 10, 14, 15 |
| State the data sources in the order of procedures used in the design type (e.g., qualitative sources first in an exploratory sequential design followed by quantitative sources), if a sequenced design is used in the mixed methods study. | We have reported accordingly. |
| Describe the qualitative and the quantitative sampling in separate sections. | 8, 9, 13 |
| Discuss the recruitment strategy for qualitative and quantitative research separately. | 8, 9, 13 |
| Devote separate sections to the qualitative data analysis, the quantitative data analysis, and the mixed methods analysis. | 12, 17, 18 |
| Indicate methodological integrity, quantitative validity and reliability, and mixed methods validity or legitimacy. | 18 |
| **Results** |  |
| Indicate how the qualitative and quantitative results were “mixed” or integrated (e.g., discussion; tables of joint displays; graphs; data transformation in which one form of data is transformed to the other, such as qualitative text, codes, themes are transformed into quantitative counts or variables). | N/A (protocol manuscript) |

**Journal Article Reporting Standards for Qualitative Research (JARS-Qual) checklist.**

| **Item** | **Page #** |
| --- | --- |
| **Title** |  |
| Identify key issues/topic under consideration. | 1 |
| Acknowledge funding sources or contributors. | 21 (per journal) |
| Acknowledge conflicts of interest, if any. | 21 (per journal) |
| **Abstract** |  |
| State the problem/question/objectives under investigation. | 2 |
| Indicate the study design, including types of participants or data sources, analytic strategy, main results/findings, and main implications/significance | 2-3 |
| Identify five keywords. | 3 |
| **Introduction** |  |
| Frame the problem or question and its context. | 4, 5 |
| Review, critique, and synthesize the applicable literature to identify key issues/debates/theoretical frameworks in the relevant literature to clarify barriers, knowledge gaps, or practical needs. | 4, 5 |
| State the purpose(s)/goal(s)/aim(s) of the study. | 5, 6 |
| State the target audience, if specific. | N/A |
| Provide the rationale for fit of design used to investigate this purpose/goal. | 7, 8 |
| Describe the approach to inquiry if it illuminates the objectives and research rationale. | 8 |
| **Methods** |  |
| Summarize the research design, including data-collection strategies, data-analytic strategies, and, if illuminating, approaches to inquiry. | 8 |
| Provide the rationale for the design selected. | 8 |
| Describe the researchers’ backgrounds in approaching the study, emphasizing their prior understandings of the phenomena under study | 6 |
| Provide the numbers of participants/documents/events analyzed. | N/A (protocol) |
| Describe the demographics/cultural information, perspectives of participants, or characteristics of data sources that might influence the data collected. | N/A (protocol) |
| Describe existing data sources, if relevant. | N/A (protocol) |
| Provide data repository information for openly shared data, if applicable. | N/A (protocol) |
| Describe archival searches or process of locating data for analyses, if applicable. | N/A |
| Describe the relationships and interactions between researchers and participants relevant to the research process and any impact on the research process. | N/A (protocol) |
| Describe the recruitment process (e.g., face-to-face, telephone, mail, email) and any recruitment protocols. | 8, 9 |
| Describe any incentives or compensation, and provide assurance of relevant ethical processes of data collection and consent process as relevant. | 12, 13 |
| Describe the process by which the number of participants was determined in relation to the study design. | 10 |
| Provide any changes in numbers through attrition and final number of participants/sources. | N/A (protocol) |
| Describe the rationale for decision to halt data collection. | N/A (protocol) |
| Describe the participants/data source selection process. | 8, 10 |
| Provide the general context for the study. | 8 |
| If your participant selection is from an archived data set, describe the recruitment and selection process from that data set as well as any decisions in selecting sets of participants from that data set. | N/A |
| State the form of data collected. | 8 |
| Describe the origins or evolution of the data-collection protocol. | N/A (protocol) |
| Describe any alterations of data-collection strategy in response to the evolving findings or the study rationale. | N/A (protocol) |
| Describe the data-selection or data-collection process (e.g., were others present when data were collected, number of times data were collected, duration of collection, context). | 10, 11 |
| Convey the extensiveness of engagement (e.g., depth of engagement, time intensiveness of data collection). | 10, 11 |
| For interview and written studies, indicate the mean and range of the time duration in the data-collection process. | N/A (protocol) |
| Describe the management or use of reflexivity in the data-collection process, as it illuminates the study. | N/A (protocol) |
| Describe questions asked in data collection: content of central questions, form of questions. | Additional File 2 |
| Describe the methods and procedures used and for what purpose/goal. | 12 |
| Explicate in detail the process of analysis, including some discussion of the procedures (e.g., coding, thematic analysis) following a principle of transparency. | 12 |
| Describe coders or analysts and their training, if not already described in the researcher description section (e.g., coder selection, collaboration groups). | 10 |
| Identify whether coding categories emerged from the analyses or were developed a priori. | 12 |
| Identify units of analysis (e.g., entire transcript, unit, text) and how units were formed, if applicable. | 12 |
| Describe the process of arriving at an analytic scheme, if applicable. | N/A |
| Provide illustrations and descriptions of the analytic scheme development, if relevant. | N/A |
| Indicate software, if used. | N/A |
| Methodological integrity | N/A (protocol) |
| **Results** | N/A (protocol) |
| **Discussion** | N/A (protocol) |

**Standard Protocol Items: Recommendations for Interventional Trials (SPIRIT) checklist.**

| **Section/Item** | **Description** | **Page #** |
| --- | --- | --- |
| **Administrative** |  |  |
| Title | Descriptive title identifying the study design, population, interventions, and, if applicable, trial acronym | 1 |
| Trial registration | Trial identifier and registry name. If not yet registered, name of intended registry | 3 |
| Protocol version | Date and version identifier | N/A |
| Funding | Sources and types of financial, material, and other support | 21 |
| Roles and responsibilities | Names, affiliations, and roles of protocol contributors | 22 |
|  | Name and contact information for the trial sponsor | N/A |
|  | Role of study sponsor and funders if any | N/A |
|  | Composition, roles, and responsibilities of the coordinating centre, steering committee, endpoint adjudication committee, data management team, and other individuals or groups overseeing the trial, if applicable | 6 |
| **Introduction** |  |  |
| Background and  rationale | Description of research question and justification for undertaking the trial, including summary of relevant studies (published and unpublished) examining benefits and harms for each intervention | 4, 5 |
|  | Explanation for choice of comparators | N/A |
| Objectives | Specific objectives or hypotheses | 6 |
| Trial design | Description of trial design including type of trial (eg, parallel group, crossover, factorial, single group), allocation ratio, and framework (eg, superiority, equivalence, noninferiority, exploratory) | 7 |
| **Methods** |  |  |
| Study setting | Description of study settings (eg, community clinic, academic hospital) and list of countries where data will be collected. | 8, 13 |
| Eligibility criteria | Inclusion and exclusion criteria for participants. If applicable, eligibility criteria for study centres and individuals who will perform the interventions (eg, surgeons, psychotherapists) | 8, 13 |
| Interventions | Interventions for each group with sufficient detail to allow replication, including how and when they will be administered | 16 |
|  | Criteria for discontinuing or modifying allocated interventions for a given trial participant | N/A (not clinical intervention) |
|  | Strategies to improve adherence to intervention protocols, and any procedures for monitoring adherence | N/A (not clinical intervention) |
|  | Relevant concomitant care and interventions that are permitted or prohibited during the trial | N/A (not clinical intervention) |
| Outcomes | Primary, secondary, and other outcomes, including the specific measurement variable (eg, systolic blood pressure), analysis metric (eg, change from baseline, final value, time to event), method of aggregation (eg, median, proportion), and time point for each outcome. Explanation of the clinical relevance of chosen efficacy and harm outcomes is strongly recommended | 8, 16, 17 |
| Participant timeline | Time schedule of enrolment, interventions (including any run-ins and washouts), assessments, and visits for participants. A schematic diagram is highly recommended | Table 2 |
| Sample size | Estimated number of participants needed to achieve study objectives and how it was determined, including clinical and statistical assumptions supporting any sample size calculations | 10, 15 |
| **Assignment of interventions (for controlled trials)** |  | **N/A** |
| **Methods: Data collection, management, and analysis** |  |  |
| Data collection methods | Plans for assessment and collection of outcome, baseline, and other trial data, including any related processes to promote data quality (eg, duplicate measurements, training of assessors) and a description of study instruments (eg, questionnaires, laboratory tests) along with their reliability and validity, if known. Reference to where data collection forms can be found, if not in the protocol | 10, 15, 16 |
|  | Plans to promote participant retention and complete follow-up, including list of any outcome data to be collected for participants who discontinue or deviate from intervention protocols | 15 |
| Data management | Plans for data entry, coding, security, and storage, including any related processes to promote data quality (eg, double data entry; range checks for data values). Reference to where details of data management procedures can be found, if not in the protocol | 11, 17 |
| Statistical methods | Statistical methods for analysing primary and secondary outcomes. Reference to where other details of the statistical analysis plan can be found, if not in the protocol | 12, 17 |
|  | Methods for any additional analyses (eg, subgroup and adjusted analyses) | N/A |
|  | Definition of analysis population relating to protocol non-adherence (eg, as randomised analysis), and any statistical methods to handle missing data (eg, multiple imputation) | N/A |
| **Methods: monitoring** |  |  |
| Data monitoring | Composition of data monitoring committee (DMC); summary of its role and reporting structure; statement of whether it is independent from the sponsor and competing interests; and reference to where further details about its charter can be found, if not in the protocol. Alternatively, an explanation of why a DMC is not needed | N/A (not a clinical intervention) |
|  | Description of any interim analyses and stopping guidelines, including who will have access to these interim results and make the final decision to terminate the trial | N/A (not a clinical intervention) |
| Harms | Plans for collecting, assessing, reporting, and managing solicited and spontaneously reported adverse events and other unintended effects of trial interventions or trial conduct | N/A (not a clinical intervention) |
| Auditing | Frequency and procedures for auditing trial conduct, if any, and whether the process will be independent from investigators and the sponsor | N/A (not a clinical intervention) |
| **Ethics and dissemination** |  |  |
| Research ethics  approval | Plans for seeking research ethics committee/institutional review board (REC/IRB) approval | 7 |
| Protocol amendments | Plans for communicating important protocol modifications (eg, changes to eligibility criteria, outcomes, analyses) to relevant parties (eg, investigators, REC/IRBs, trial participants, trial registries, journals, regulators) | N/A (short term study) |
| Consent or assent | Who will obtain informed consent or assent from potential trial participants or authorised surrogates, and how | 8, 9, 14 |
|  | Additional consent provisions for collection and use of participant data and biological specimens in ancillary studies, if applicable | N/A |
| Confidentiality | How personal information about potential and enrolled participants will be collected, shared, and maintained in order to protect confidentiality before, during, and after the trial | 12 |
| Declaration of interests | Financial and other competing interests for principal investigators for the overall trial and each study site | 22 |
| Access to data | Statement of who will have access to the final trial dataset, and disclosure of contractual agreements that limit such access for investigators | N/A |
| Ancillary and post-trial care | Provisions, if any, for ancillary and post-trial care, and for compensation to those who suffer harm from trial participation | N/A |
| Dissemination policy | Plans for investigators and sponsor to communicate trial results to participants, healthcare professionals, the public, and other relevant groups (eg, via publication, reporting in results databases, or other data sharing arrangements), including any publication restrictions | 19 |
|  | Authorship eligibility guidelines and any intended use of professional writers | N/A |
|  | Plans, if any, for granting public access to the full protocol, participant level dataset, and statistical code | N/A |
| **Appendices** |  |  |
| Informed consent materials | Model consent form and other related documentation given to participants and authorised surrogates | Additional file 1 |
| Biological specimens | Plans for collection, laboratory evaluation, and storage of biological specimens for genetic or molecular analysis in the current trial and for future use in ancillary studies, if applicable | N/A |

**Guideline for reporting health research design checklist.**

| Section/Item | Description | Page # |
| --- | --- | --- |
| Title | Title should indicate that the study included a design approach | 1 |
| Abstract | Abstract summarises the salient components, including background, statement of the problem, approach/ methods, findings, results and conclusion | 2, 3 |
| Introduction/background | Overview of the background to the topic, what has been done in the area already and rationale for using the design | 4, 5 |
| Available knowledge | Summary of what is known about the topic and any gaps | 4, 5 |
| Rationale for design approach | Explanation of design as appropriate to address the topic | 6 |
| Description of design challenge for health | Description of the main research question or health problem that the design-based work aimed to address | 5 |
| Research aims | Overall aims and objectives of the project | 6 |
| Methods/approach | Description of approach used for the research | 6 |
| Theory | Underlying theories that informed this work (if any) | N/A |
| Process and timeline | Processes or steps and timeline for the research | Figure 1, table 2 |
| Research team characteristics and reflexivity | Individuals involved in the research team and characteristics of these. Ways that reflexivity was addressed | 6 |
| Site selection | Description of study sites selected | N/A |
| Participant selection and engagement | Process to select participants, description of the participants and explanation of how they were involved in design | 8, 9 |
| Ethical considerations | Ethical precautions taken to protect participants, communities, and personal information | 12 |
| Language | Description of any language considerations | 19 |
| Techniques to understand (data collection tools and instruments) | Techniques or tools used during the research process | 10, 11 |
| Documentation | Documentation of work undertaken in research. | 10, 11 |
| Techniques to synthesise | Description of techniques used to synthesise insights, iterate, and analyse data | 12 |
| Validation approaches | Process of checking that insights, prototypes or other products were validated. | 10 |
| Results/findings from design research and activities | Summary of findings from design activities, resulting insights, what was designed, what resulted from the work and (if available) impact of activities; report on any secondary or ancillary results | N/A (protocol) |
| Design research phase | Summary of major insights or reflection from design activities | N/A (protocol) |
| Decision points | Description of decisions made during the design process | N/A (protocol) |
| Evidence of change or impact | Summary of any evidence of change or impact to health or other facets of the situation | N/A (protocol) |
| Discussion | Reflection on design as an approach to the health topic and the strengths and limitations of the work | N/A (protocol) |
| Design | Reflection of the application of design to this research topic | N/A (protocol) |
| Conclusion | Implications of this work for the larger field and next steps | N/A (protocol) |
| Other | Acknowledgements of support, assistance, funding, statement on conflict of interest and authors’ contributorship | 21, 22 |
| Glossary | Definition of key terms | N/A (protocol) |

**Phase one letter of information.**

Patient/substitute decision maker version:

Dear Participant (Address participant by first and last name)

Thank you for your interest in joining the Canadian Adaptive Platform Trial in Intensive Care (CAPTIC) patient panel.

We are currently seeking members of the CAPTIC patient panel to participate in a research study involving focus group discussions. The [CAPTIC Research Program](http://www.captic.ca/), based out of St. Michael's Hospital, Unity Health Toronto is working to improve the consenting process to ICU research for patients, families and other individuals involved in this process. As part of the improvement efforts, the CAPTIC team has created a few digital or paper-based prototypes of a resource for supporting the consent process. As part of this research study, you would be providing your experience and feedback on these prototypes within a focus group with other participants

This focus group is completely voluntary - if you are not interested in participating, you may decline, and you will still be invited to participate in future patient panel activities. If you are interested, we will follow up with an email with potential dates and times for focus group activities and ask that you please kindly let us know your availability.

As a participant, you are invited to join in a few activities to help improve the resource:

1. **Focus Group Pre-work**
   This pre-work involves background readings and a small note-taking activity in preparation for the focus group. You will be completing the pre-work individually and offline. The pre-work may take 1.5–2 hours to complete.
2. **Resource Design Focus Group**
   You will be reviewing the resource prototypes in a Zoom meeting and sharing your feedback and experience in a discussion with a group of 4-6 patients/substitute decision makers and research coordinators. The meeting will be approximately 2 hours in length.
3. **Review Meeting**
   The research team will be refining the resource based on the feedback collected in the focus group and presenting the updated resource in this post-workshop Zoom meeting. You will be invited to share your last feedback on the updated resource. The meeting will be approximately 1 hour in length.

We understand that participation in this focus group will involve discussing sensitive topics and may re-ignite or worsen feelings of stress, anxiety and/or grief, which are typical of an ICU encounter. As participants, though your full commitment is highly encouraged, you will not have to answer all questions and you may also skip questions which you feel you do not want to answer. You may also pause or leave or exit the focus group at any time.

Lastly, though the focus group discussions will be audio-recorded, all information shared during the workshops will be kept confidential. We also ask that you keep what is shared during the workshop confidential; however, we cannot guarantee that other participants will not share your information or responses. Direct quotes from your responses during the workshop may be used in reports or publications, but the quotes will not be attributed to you or contain any information that could be used to identify you. Your consent to participate will be implied by participating in the workshops.

If we do not receive response from you, we will follow up with you once, five-days after this email. If you do not wish to receive a follow-up, you may respond to this email indicating so.

Thank you very much for your consideration and we look forward to having you join our research study.

The CAPTIC Research Team

(Include PI Names)

Research coordinator version:

Dear Participant (Address participant by first and last name)

We are currently seeking REMAP-CAP research coordinators to participate in a consent research study involving focus group discussions. The [CAPTIC Research Program](http://www.captic.ca/), based out of St. Michael's Hospital, Unity Health Toronto is working to improve the consenting process to ICU research for patients, families and other individuals involved in this process. As part of the improvement efforts, the CAPTIC team has created a few digital or paper-based prototypes of a resource for supporting the consent process. As part of this research study, you would be providing your experience and feedback on these prototypes within a focus group with other participants

This focus group is completely voluntary - if you are not interested in participating, you may decline. If you are interested, we will follow up with an email with potential dates and times for focus group activities and ask that you please kindly let us know your availability.

As a participant, you are invited to join in a few activities to help improve the resource:

1. **Focus Group Pre-work**
   This pre-work involves background readings and a small note-taking activity in preparation for the focus group. You will be completing the pre-work individually and offline. The pre-work may take 1.5–2 hours to complete.
2. **Resource Design Focus Group**
   You will be reviewing the resource prototypes in a Zoom meeting and sharing your feedback and experience in a discussion with a group of 4-6 patients/substitute decision makers and research coordinators. The meeting will be approximately 2 hours in length.
3. **Review Meeting**
   The research team will be refining the resource based on the feedback collected in the focus group and presenting the updated resource in this post-workshop Zoom meeting. You will be invited to share your last feedback on the updated resource. The meeting will be approximately 1 hour in length.

As participants, though your full commitment is highly encouraged, you will not have to answer all questions and you may also skip questions which you feel you do not want to answer. You may also pause or leave or exit the focus group at any time.

Lastly, though the focus group discussions will be audio-recorded, all information shared during the workshops will be kept confidential. We also ask that you keep what is shared during the workshop confidential; however, we cannot guarantee that other participants will not share your information or responses. Direct quotes from your responses during the workshop may be used in reports or publications, but the quotes will not be attributed to you or contain any information that could be used to identify you. Your consent to participate will be implied by participating in the workshops.

If we do not receive response from you, we will follow up with you once, five-days after this email. If you do not wish to receive a follow-up, you may respond to this email indicating so.

Thank you very much for your consideration and we look forward to having you join our research study.

The CAPTIC Research Team

(Include PI Names)

**Phase two letter of information.**


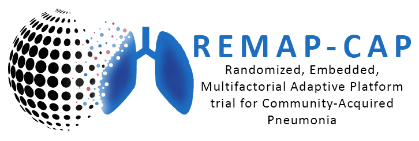


Insert Institutional Logo

**Information Letter for a survey questionnaire – Patient/SDM Version**

Dear Patient/SDM,

When you or your loved one were admitted to the ICU at [Insert hospital name here], you were invited to participate in a research study called REMAP-CAP. This is a research study looking at different ways to identify the effect of a range of strategies to improve survival, recovery and reduce the time a patient with pneumonia spends in the Intensive Care Unit and hospital.

We are interested in improving the REMAP-CAP consent experience for future patients and families. To do this, we are conducting a small pilot research study, titled “Pilot Testing an Infographic to Support Patients/Families through the REMAP-CAP Consent Process “. Our research study involves asking you, if you are able, to complete an electronic survey, which will take about 10-15 minutes to complete. The survey will tell us about your experience when you or your loved one were asked to participate in REMAP-CAP. We anticipate receiving feedback from up to 60 patients/family members from five REMAP-CAP study sites in Ontario, Canada.

You do not have to complete the survey if you do not want to. This feedback is voluntary. You can decline to participate, or choose to answer some, or all of the questions. Your responses will not be included unless you hit “submit” on the final page of the survey. We understand that participation in this survey may re-ignite or worsen feelings of stress, anxiety and/or grief, which are typical of an ICU encounter, in which case you may choose to pause or stop participation in the survey. The research study team is also available to discuss any concerns and/or to refer you to available resources (contact information below). This will not adversely affect your future care or relationship with staff at [insert hospital name here]. The information you provide will remain confidential. Your data will only be linked to you through a master list, which will be stored by the Research Coordinator at [insert hospital name here]. Hard copies of the survey will be stored in a locked filing cabinet, in a locked office and electronic copies will be password-protected and stored on the hospital’s secure server. Your name will not appear anywhere, and your data will be grouped with survey participants. Direct quotes from your responses to the survey may be used in reports or publications, but the quotes will not be attributed to you or contain any information that could be used to identify you. The data collected through this survey will be kept for a period of at least 7 years in a secure location and then destroyed. You may choose to withdraw your survey responses at any time by contacting the research study investigators. Your response will be deleted from the database.

Should you choose to participate, you will be completing the survey using LimeSurvey, a web-based secure platform for survey administration. LimeSurvey’s servers are located in Hamburg, Germany. We will not collect or use Internet Protocol (IP) addresses or any other information which could link your participation to your electronic device. No personal identifiers will be entered in LimeSurvey. Your name and email will be shared with the research team (jointly coordinated at McMaster University and St. Michael’s Hospital). The survey link will be sent to you by email. There are common risks of using email to communicate:

- Information travels electronically and is not secure in the way a phone call or regular mail would be.
- If someone sees these emails, they may know that you are a participant in this research study.
- Emails may be read or saved by your internet or phone providers (i.e. Rogers, your workplace, “free internet” providers).
- Copies of an email may continue to exist, even after efforts to delete the email have been made.
- There is always a chance with any unencrypted email, however remote, that is could be intercepted or manipulated.

To protect your privacy, your email will be stored separately from your survey responses. A master linking log containing email information will be created for this purpose. This means that your survey responses will be de-identified and can only be linked to you with the master log.

If you are unable to access email or complete the electronic questionnaire, you may choose to complete a paper copy of the survey. You will be asked to return the completed survey to the ICU research office at [insert room number here].

Your decision to participate in this survey will not impact your participation in REMAP-CAP. All participants will receive a $5 gift card to recognize their time and important contributions to this pilot research study. Participants will receive a gift card regardless of how many survey questions they choose to answer.

This survey of your consent experience has been reviewed through Clinical Trials Ontario (CTO ID XXXX). If you have questions for the Research Board that reviewed the survey, contact the Unity Health Toronto Research Ethics Board at 416-864-6060 ext. 42557.

For all other questions about the survey, please feel free to contact the Research Coordinator at [insert telephone number here].

Thank you for your assistance with helping us learn more about your experience.

Yours sincerely,

The REMAP-CAP Trial Team

Research Coordinator Contact Information:

Name: ________________________________

Telephone: ____________________________

I have read the information presented in the information letter about a survey being conducted to improve the REMAP-CAP consent experience for patients and families. I have had the opportunity to ask any questions related to this survey, to receive satisfactory answers to my questions, and any additional details I wanted. I am aware that I choose to answer some, or all of the survey questions.

**□ Yes, I agree to have a member of the research team follow up with me to ask for my feedback in a short survey.**

**Email address (if applicable): ______________________________**

**_____________________ ________________ ___________________**

**Name of Participant/SDM Signature Date**

**_______________________________**

**Witness (if applicable)**

□ No, I don’t agree to have a member of the research team follow up with me to ask for my feedback in a short survey.

**Stakeholder review meeting agenda and overview.**

Objectives for the Final Review meeting: To collect final thoughts and feedback on the revised infographic from Patients/SDMs + RCs (primary), PFP (secondary) on areas below to inform one last minor iteration of the RCT before final delivery:

1. Comparing if revised RCT is easier to understand than previous version from workshop
2. Identifying features within the RCT that may prevent/deter/limit a person's use of the RCT

Purpose of each group's attendance:

- Research team (silent observers): to observe live patient feedback on the RCT
- PFP: to see the amalgamation of the draft RCTs and design activities and to provide last feedback on the revised RCT in response to scripted questions
- Patients/SDMs and RCs: to see the outcome of the draft RCTs from the workshop activities and to provide last feedback on the revised RCT in response to scripted questions
- Additional research team member:
  - to act as the meeting's co-facilitator
  - to record, digitally (audio), the feedback discussion

In terms of potential number of attendees for this meeting, we may have up to:

- 2 facilitators
- Silent observers
  - 5+ research team members/RCs - attendance can be optional for those research team members who were not silent observers during the workshops
  - 3 PFPs
- Active participants
  - 6 Patients/SDMs and 4 RCs

Rough agenda:

1. Welcome all attendees; share context of where we are in the project (e.g. the key design activities relevant to the PFP, Patients/SDMs and RCs)
2. Share workshop learnings
3. Share revised RCT and design rationale
4. Ask PFP, Patients/SDMs and RCs scripted questions while second facilitator records
5. Close out meeting; share any next steps

Example questions:

- Do you feel that having this RCT would make the consent process easier to understand than without (when you only have the consent form and domain appendices)?
- Do you feel that this revised RCT is easier to understand compared to the versions you saw in the workshop?
- Are there any small changes that you would suggest that would have a big value in making the RCT better (in terms of comprehension, ease of use, approachability, etc.)?
