# Supplementary material for: Co-designing and pilot testing an infographic to support patients/families through the REMAP-CAP consent process: a mixed-methods study protocol
Source: Pilot Feasibility Stud. 2023 Apr 13;9:58. doi: 10.1186/s40814-023-01290-6 (PMC10098229; doi:10.1186/s40814-023-01290-6)
Supplement: Supplementary file 2 — Additional file 2. [file 40814_2023_1290_MOESM2_ESM.pdf]

Introduction to the REMAP-CAP Trial

REMAP-CAP is a platform trial which is a new form of clinical research evaluating multiple treatment interventions at the same time. Interventions are separated into domains.

- If eligible for participation, you will be randomized to receive interventions in one or more of the domains.
- Interventions are analyzed as the trial progresses.
- If patients are found to be doing better based on results in particular domains, then new patients are more likely to be randomized to these better performing domains.

REMAP-CAP continuously evaluates the interventions to determine the best plan to treat community-acquired pneumonia, including COVID-19.

Domain	COVID-19 Interventions	Community-acquired Pneumonia Interventions
**Antibiotics**	• Ceftriaxone + azithromycin<br>• Piperacillin-tazobactum + azithromycin<br>• Levofloxacin or Moxifloxacin	• Ceftriaxone + azithromycin<br>• Piperacillin-tazobactum + azithromycin<br>• Levofloxacin or Moxifloxacin
**Azithromycin Duration**	• Azithromycin for 3 to 5 days<br>• Azithromycin for 14 days	• Azithromycin for 3 to 5 days<br>• Azithromycin for 14 days
**Antivirals**	• No Oseltamivir<br>• Oseltamivir for 5 days<br>• Oseltemavir for 10 days	• No Oseltamivir<br>• Oseltamivir for 5 days<br>• Oseltemavir for 10 days
**Anticoagulants**	• Low dose<br>• Medium dose<br>• Full dose	*
**Corticosteroids**	*	• No corticosteroid<br>• Fixed duration hydrocortisone for 7 days<br>• Hydrocortisone given only when in septic sock
**Statins**	• No Simvastatin<br>• Simvastatin for up to 28 days	*

* Not available

Glossary of Terms

Patients with pneumonia, including SARS CO-V (COVID-19) who are eligible, will be randomized to receive one intervention in one, or more, of the trial treatment domains. All patients will receive the best standard of care.

Watch a quick video on how the trial works by scanning this QR code with your phone!

SCAN ME

**Antibiotics**	Medicines that kill bacteria
**Anticoagulants**	Medicines that prevent blood from forming clots (eg. Heparin)
**Antivirals**	Medicines that prevent the virus from multiplying
**Corticosteroids**	Medicines that reduce inflammation (eg. cortisone)
**Domain**	A set of treatment options within a single clinical area such as blood clotting or inhibiting the virus
**Intervention**	A specific treatment option within a domain
**Azithromycin Duration**	The duration of time for which the antibiotic, Azithromycin, is given
**Randomization**	A method of selection based on chance alone by which participants are assigned to a treatment group in a domain
**REMAP-CAP**	Randomized Embedded Multi-factorial Adaptive Platform Trial for Community-Acquired Pneumonia (including the SARS coronavirus: COVID-19)
**Statins**	Medicines that lower cholesterol, and may reduce inflammation

To learn more about the trial, visit **www.remapcap.org**

Your Guide to the REMAP-CAP Study

A research team is inviting you or your loved one to join the REMAP-CAP study. This study is for patients in the ICU with pneumonia or suspected or confirmed COVID-19.

Research Contact:

Contact Info:

Consent Follow-up:

How the study works

1 REMAP-CAP uses a menu with different **sections.** Each section has many **items** in it. Each item is a medication or treatment that might be able to treat a condition.

This study can test more than one item at a time.

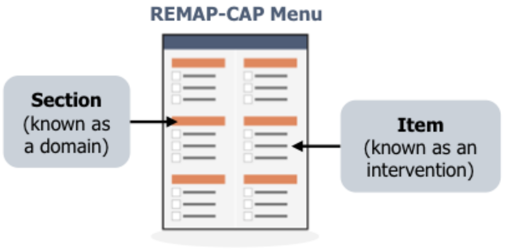

2 Your healthcare team has updated the menu for you on the back of this page. **Your menu shows the sections that are most suitable for your current condition.**

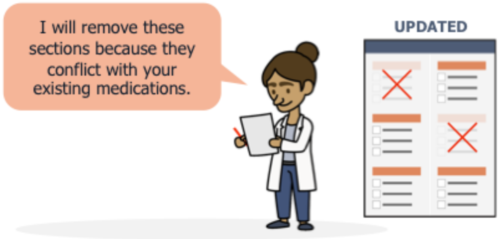

3 Your research contact will go over each section with you and answer your questions. **Make sure to tell them if you are uncomfortable with anything for any reason.** They will update the menu based on your discussion.

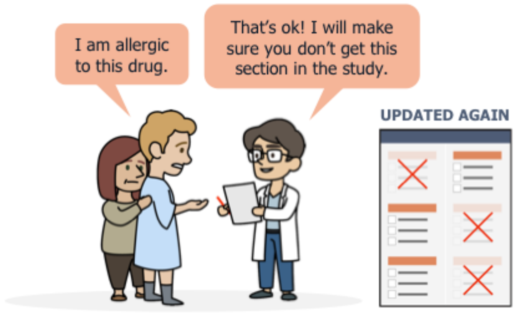

4 Your healthcare team will choose one or more sections left on your menu. Then a computer will choose one item from each of these sections.

These will be the items you will get in the study.

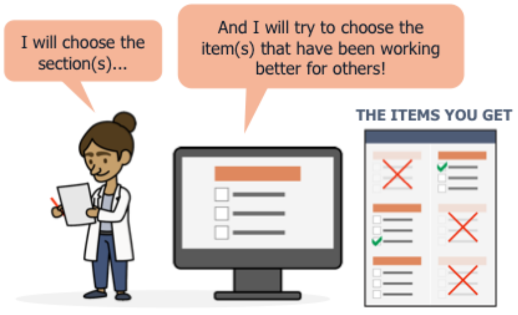

Your participation will not affect the quality of your care

You get to decide if you want to join the study. You can also leave the study at any time after joining.

- If you choose to join, you will be helping to improve care for future patients.
- If you choose not to join, you will still receive the best standard of care.

Your Menu

P For pneumonia   C For COVID-19

Antibiotics P C

Antibiotics are widely used in usual care to treat infections caused by bacteria. COVID-19 is caused by a virus but can lead to bacterial infections.

INTERVENTIONS

REMAP-CAP studies which combinations of antibiotics are most effective in treating pneumonia and/or COVID-19.

- ❑ Ceftriaxone + azithromycin
- ❑ Piperacillin-tazobactam + azithromycin
- ❑ Levofloxacin or moxifloxacin

HOW YOU GET IT

Through mouth, feeding tube, or a thin tube inserted into your vein (called an intravenous).

Azithromycin Duration P C

Azithromycin is an antibiotic that is widely used in usual care to reduce inflammation. Duration refers to the length of the azithromycin treatment.

INTERVENTIONS

REMAP-CAP studies what azithromycin duration is most effective in treating pneumonia and/or COVID-19.

- ❑ Azithromycin for 3 to 5 days
- ❑ Azithromycin for 14 days

HOW YOU GET IT

Through mouth, feeding tube, or a thin tube inserted into your vein (called an intravenous).

Antivirals P C

Antivirals are widely used in usual care to treat infections caused by some but not all viruses.

INTERVENTIONS

Oseltamivir is an antiviral. REMAP-CAP studies which oseltamivir option is most effective in treating pneumonia suspected to be caused by the influenza virus.

- ❑ Oseltamivir for 5 days
- ❑ Oseltamivir for 10 days
- ❑ No oseltamivir

HOW YOU GET IT

Through mouth or feeding tube.

Blood Thinners C

Blood thinners are widely used in usual care to prevent or reduce blood clots. COVID-19 can lead to blood clots and inflammation.

INTERVENTIONS

REMAP-CAP studies whether blood thinners are effective in treating COVID-19. It is not known how much blood thinners should be given in COVID-19 or for how long.

- ❑ Low dose
- ❑ Medium dose
- ❑ Full dose

HOW YOU GET IT

Through a thin tube inserted into your vein (called an intravenous) or injected just under the skin.

Corticosteroids P

Corticosteroids are widely used in usual care to reduce inflammation.

INTERVENTIONS

Hydrocortisone is a corticosteroid. REMAP-CAP studies which hydrocortisone option is most effective in treating pneumonia.

- ❑ Hydrocortisone for 7 days
- ❑ Hydrocortisone given only when in septic shock (i.e. severe infection)
- ❑ No corticosteroid

HOW YOU GET IT

Through a thin tube inserted into your vein (called an intravenous).

Statins C

Statins are widely used in usual care to reduce cholesterol (fats) in the blood, and may also reduce inflammation.

INTERVENTIONS

Simvastatin is a statin. REMAP-CAP studies whether simvastatin is effective in treating COVID-19.

- ❑ Simvastatin for up to 28 days
- ❑ No simvastatin

HOW YOU GET IT

Through mouth or feeding tube.

What is REMAP-CAP?

REMAP-CAP is a research trial for patients with pneumonia or suspected or confirmed COVID-19. Participation in this trial is completely voluntary. You can opt out at any time even after joining.

Interventions are medications or treatments that have the potential to treat a condition.

Traditional trials
often test only **one intervention** at a time

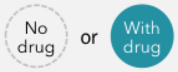

REMAP-CAP trial
tests **more than one intervention** at a time

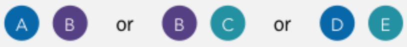

A simple way to think about REMAP-CAP:

All the interventions in the trial together is like a **deck of cards**

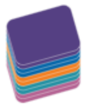

A single **intervention** is like a **card** in the deck

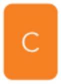

A **domain** (or category) that an intervention belongs to is like a **suit**

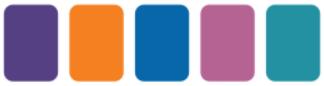

Your healthcare team has checked your medical history. They have **removed the suits that are not suitable for you** from your card deck on the back of this page. For example, you may be allergic to a drug.

Your card deck

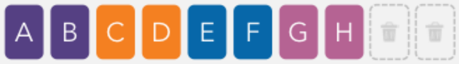

Discuss with your research coordinator if you are not comfortable with any of the suits left. They will also remove these suits from your card deck.

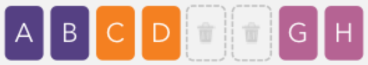

Your healthcare team will choose one or more suits left in your card deck.

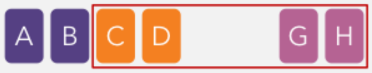

Then a computer will choose one card from each of those suits. These cards will be the interventions that you get in the trial. In this example, you will get interventions C and G.

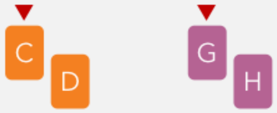

What is the difference between usual care and REMAP-CAP?

Standard care (usual care)
Standard care uses interventions (medications or treatments) that have been studied and are proven safe. Your doctor may give you one or more standard interventions even if you are not in the trial.

REMAP-CAP trial
REMAP-CAP tests many interventions used in standard care. But it may test the interventions in a new condition such as COVID-19. Or it may compare interventions to each other to see which is more effective.

What are the benefits and risks of joining the trial?

Benefits

There may or may not be direct benefits for you if you join the trial. Your data from the trial will help researchers improve care for future patients.

Risks

Each intervention has its own risks. Your research coordinator will review each intervention and its risks with you. You get to decide which interventions you are comfortable with.

Your card deck

DOMAINS	✓ INTERVENTIONS	NOTES
**Antibiotics**<br>*For pneumonia and COVID-19*<br>Antibiotics help to fight some types of infection caused by bacteria. Some viruses can also lead to bacterial infections.	☐ Ceftriaxone + azithromycin<br>☐ Piperacillin-tazobactam + azithromycin<br>☐ Levofloxacin or moxifloxacin	
**Azithromycin duration**<br>*For pneumonia and COVID-19*<br>Azithromycin is an antibiotic. Duration refers to how long you would receive azithromycin for.	☐ Azithromycin for 3 to 5 days<br>☐ Azithromycin for 14 days	
**Antivirals**<br>*For pneumonia and COVID-19*<br>Antivirals help to fight infections caused by viruses. COVID-19 is caused by a virus.	☐ No oseltamivir<br>☐ Oseltamivir for 5 days<br>☐ Oseltamivir for 10 days	
**Blood thinners**<br>*For COVID-19 only*<br>Blood thinners help to prevent or reduce blood clotting. It is not known how effective blood thinners are in treating COVID-19.	☐ Low dose blood thinner<br>☐ Intermediate dose blood thinner<br>☐ Continuation of full dose blood thinner	
**Corticosteroids**<br>*For pneumonia only*<br>Corticosteroids help to fight inflammation.	☐ No corticosteroid<br>☐ Fixed duration hydrocortisone for 7 days<br>☐ Hydrocortisone given only in septic shock	
**Statins**<br>*For COVID-19 only*<br>Statins help to reduce serum cholesterol and may reduce inflammation. It is not known how effective statins are in treating COVID-19.	☐ No simvastatin<br>☐ Simvastatin for up to 28 days	
