# Supplementary material for: Co-designing and pilot testing an infographic to support patients/families through the REMAP-CAP consent process: a mixed-methods study protocol
Source: Pilot Feasibility Stud. 2023 Apr 13;9:58. doi: 10.1186/s40814-023-01290-6 (PMC10098229; doi:10.1186/s40814-023-01290-6)

REMAP-CAP

RESOURCE DESIGN WORKSHOP PREWORK

For [workshop date]

This document is confidential. Please do not share this document with others.

INTRODUCTION

About REMAP-CAP

REMAP-CAP is a type of clinical trial currently being studied at many hospitals around the world, including St. Michael's Hospital in Toronto.

This trial is for eligible patients in the ICU who have pneumonia or suspected or confirmed COVID-19. If the patient is not in a physical or mental condition to decide for themselves, the research team will ask the patient's **substitute decision maker (SDM)** to make the consent decision on behalf of the patient. The SDM is often the patient's family member.

Below is the current REMAP-CAP **consent process**:

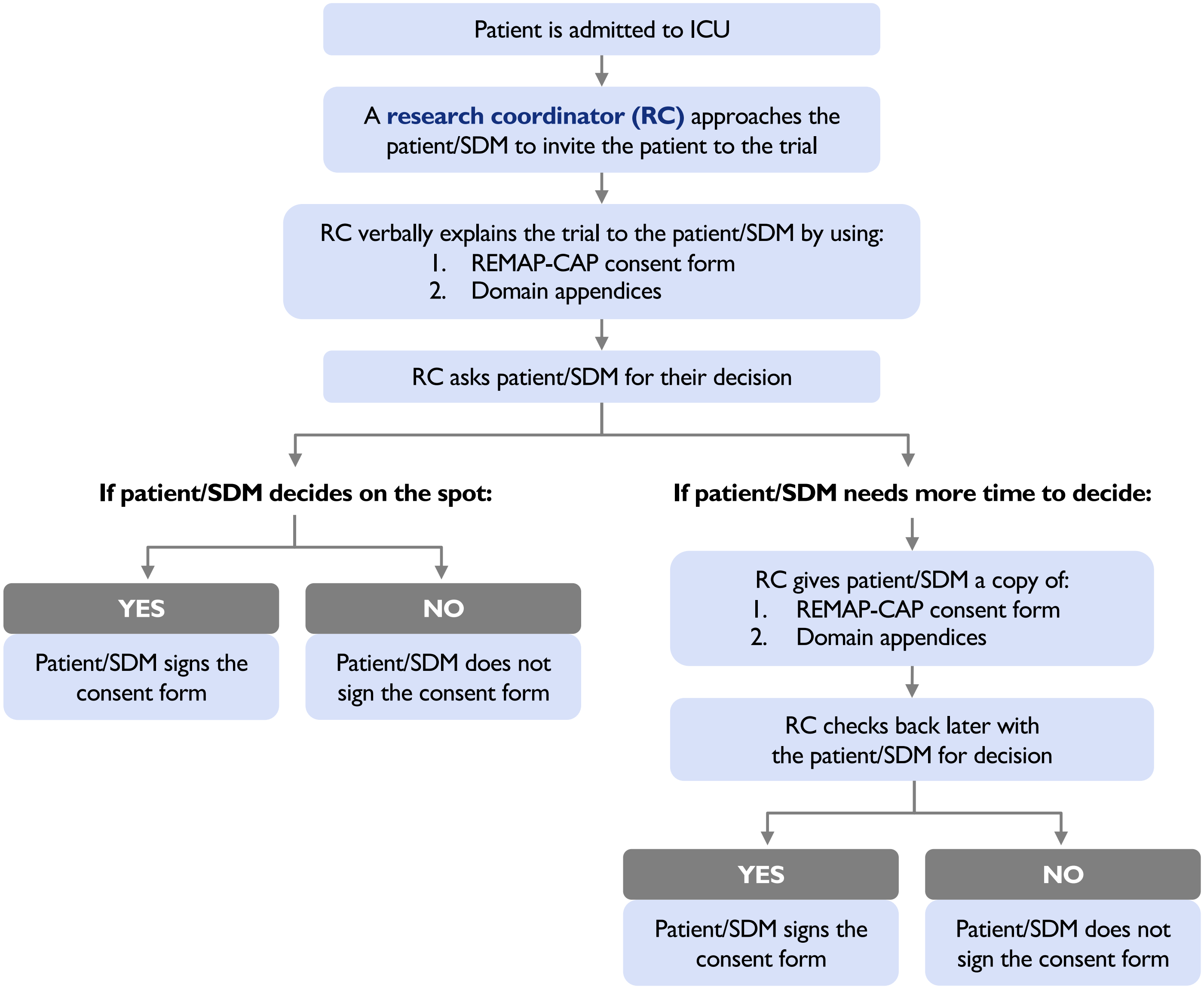

This document is confidential. Please do not share this document with others.

INTRODUCTION

Why we need your help

REMAP-CAP is a newer type of trial that is more complex.

As part of the invite, a research coordinator (RC) must explain to the patient or substitute decision maker (SDM) how the trial works. However, the complexity can be harder to explain. And it can be harder for a patient/SDM to understand. If a patient/SDM does not have a good understanding of the trial, it can be harder for them to decide.

The research team is working on making a short resource to help patients, SDMs, and RCs with this problem. They would like to add this resource to the consent process in the future:

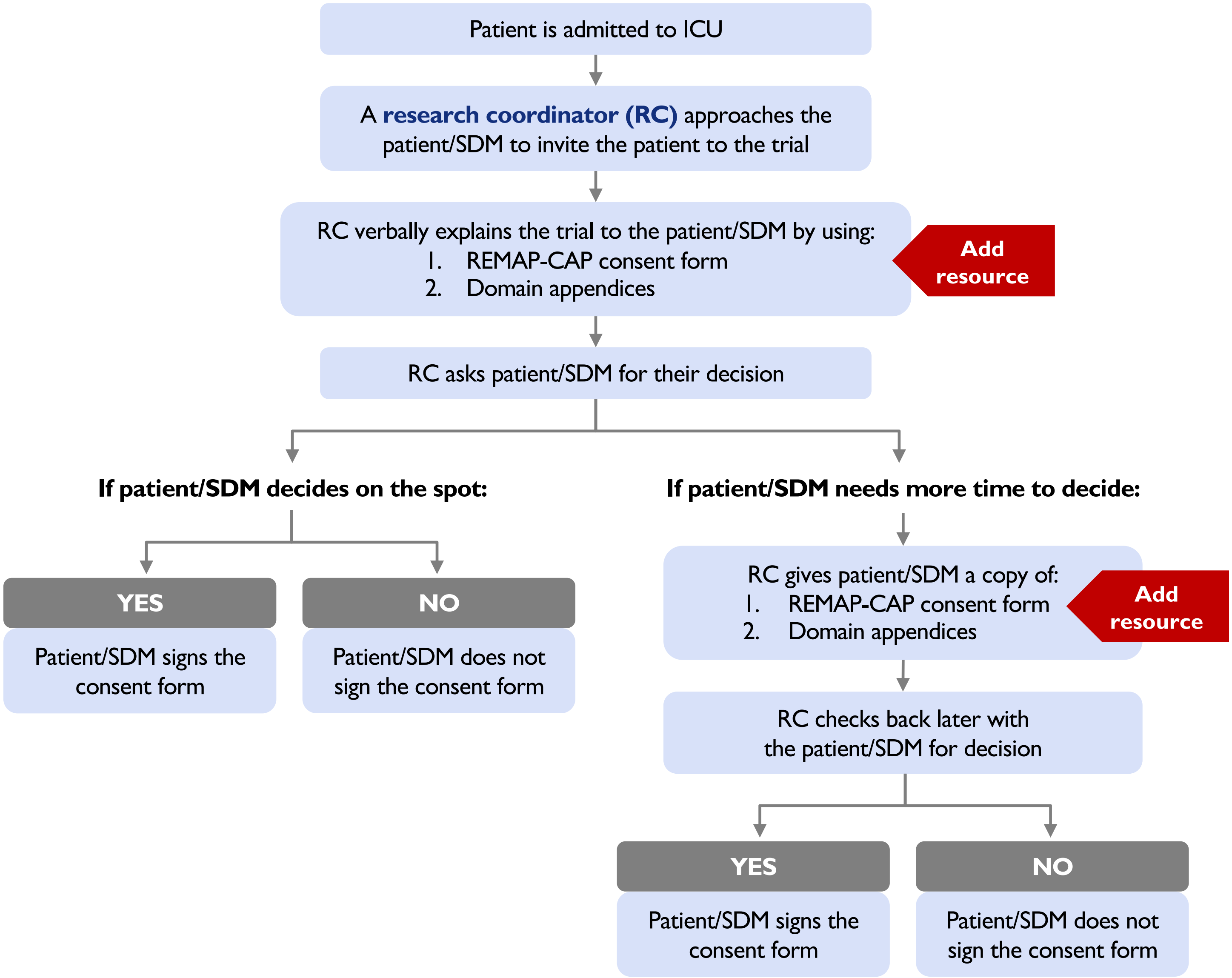

INTRODUCTION

How you can help

The research team has created 3 prototypes of the resource. The 3 prototypes are different in many ways. For example, they may contain different information or wording for the same messages.

You have been invited to this workshop as a potential user of this resource. **The team would like your feedback on the prototypes they have created.** Your feedback will help them understand how they can make this resource helpful and useful to patients/SDMs/RCs in the consent process.

IN THIS FILE

There are five sections for you to review in this file:

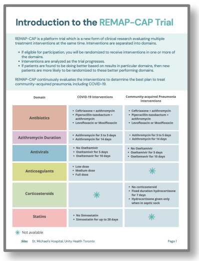

Prototype 1
(2 pages)

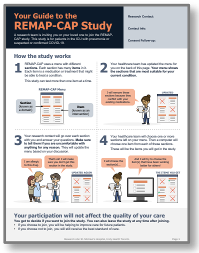

Prototype 2
(2 pages)

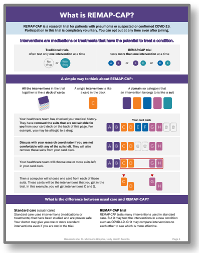

Prototype 3
(2 pages)

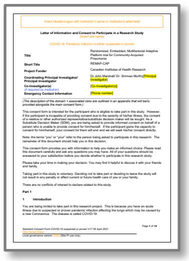

Consent Form
(14 pages)

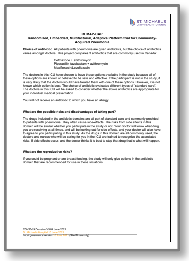

Domain Appendices
(7 pages)

BEFORE YOU START

Please grab a pen and paper so that you can write down your feedback.
You can also record your feedback digitally, if you prefer. You will be sharing your feedback with the team during the workshop.

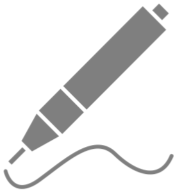

AS YOU REVIEW

Please read through the sections in order. At the beginning and end of some sections, you will be given instructions on what to do for that section.

The following **2 pages** contain

Prototype I

Please read both pages carefully.

This document is confidential. Please do not share this document with others.

Introduction to the REMAP-CAP Trial

REMAP-CAP is a platform trial which is a new form of clinical research evaluating multiple treatment interventions at the same time. Interventions are separated into domains.

- If eligible for participation, you will be randomized to receive interventions in one or more of the domains.
- Interventions are analyzed as the trial progresses.
- If patients are found to be doing better based on results in particular domains, then new patients are more likely to be randomized to these better performing domains.

REMAP-CAP continuously evaluates the interventions to determine the best plan to treat community-acquired pneumonia, including COVID-19.

Domain	COVID-19 Interventions	Community-acquired Pneumonia Interventions
**Antibiotics**	• Ceftriaxone + azithromycin<br>• Piperacillin-tazobactum + azithromycin<br>• Levofloxacin or Moxifloxacin	• Ceftriaxone + azithromycin<br>• Piperacillin-tazobactum + azithromycin<br>• Levofloxacin or Moxifloxacin
**Azithromycin Duration**	• Azithromycin for 3 to 5 days<br>• Azithromycin for 14 days	• Azithromycin for 3 to 5 days<br>• Azithromycin for 14 days
**Antivirals**	• No Oseltamivir<br>• Oseltamivir for 5 days<br>• Oseltemavir for 10 days	• No Oseltamivir<br>• Oseltamivir for 5 days<br>• Oseltemavir for 10 days
**Anticoagulants**	• Low dose<br>• Medium dose<br>• Full dose	*
**Corticosteroids**	*	• No corticosteroid<br>• Fixed duration hydrocortisone for 7 days<br>• Hydrocortisone given only when in septic sock
**Statins**	• No Simvastatin<br>• Simvastatin for up to 28 days	*

* Not available

Site: St. Michael's Hospital, Unity Health Toronto

This document is confidential. Please do not share this document with others.

Glossary of Terms

Patients with pneumonia, including SARS CO-V (COVID-19) who are eligible, will be randomized to receive one intervention in one, or more, of the trial treatment domains. All patients will receive the best standard of care.

Watch a quick video on how the trial works by scanning this QR code with your phone!

SCAN ME

**Antibiotics**	Medicines that kill bacteria
**Anticoagulants**	Medicines that prevent blood from forming clots (eg. Heparin)
**Antivirals**	Medicines that prevent the virus from multiplying
**Corticosteroids**	Medicines that reduce inflammation (eg. cortisone)
**Domain**	A set of treatment options within a single clinical area such as blood clotting or inhibiting the virus
**Intervention**	A specific treatment option within a domain
**Azithromycin Duration**	The duration of time for which the antibiotic, Azithromycin, is given
**Randomization**	A method of selection based on chance alone by which participants are assigned to a treatment group in a domain
**REMAP-CAP**	Randomized Embedded Multi-factorial Adaptive Platform Trial for Community-Acquired Pneumonia (including the SARS coronavirus: COVID-19)
**Statins**	Medicines that lower cholesterol, and may reduce inflammation

To learn more about the trial, visit **www.remapcap.org**

This document is confidential. Please do not share this document with others.

This is the end of Prototype 1.

Before moving on, imagine you received this resource as a patient in the ICU or as a substitute decision maker for a loved one in the ICU.

Please write down:
- 3 things you liked about this prototype
- 3 things you disliked about this prototype
- Overall feelings about this prototype

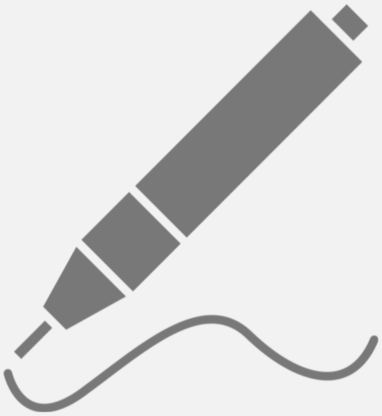

The following **2 pages** contain

Prototype 2

Please read both pages carefully.

Your Guide to the REMAP-CAP Study

A research team is inviting you or your loved one to join the REMAP-CAP study. This study is for patients in the ICU with pneumonia or suspected or confirmed COVID-19.

Research Contact:

Contact Info:

Consent Follow-up:

How the study works

1 REMAP-CAP uses a menu with different **sections.** Each section has many **items** in it. Each item is a medication or treatment that might be able to treat a condition.

This study can test more than one item at a time.

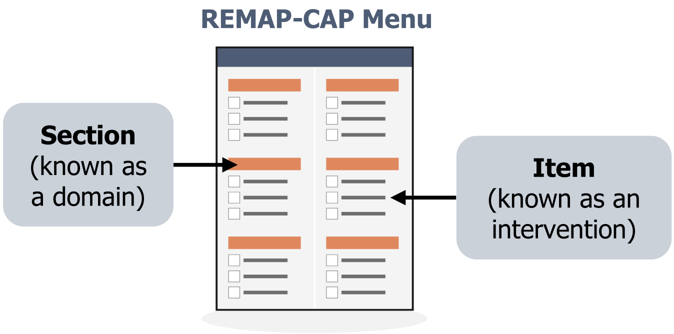

2 Your healthcare team has updated the menu for you on the back of this page. **Your menu shows the sections that are most suitable for your current condition.**

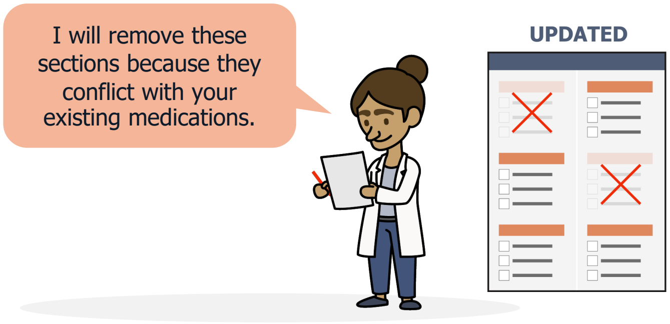

3 Your research contact will go over each section with you and answer your questions. **Make sure to tell them if you are uncomfortable with anything for any reason.** They will update the menu based on your discussion.

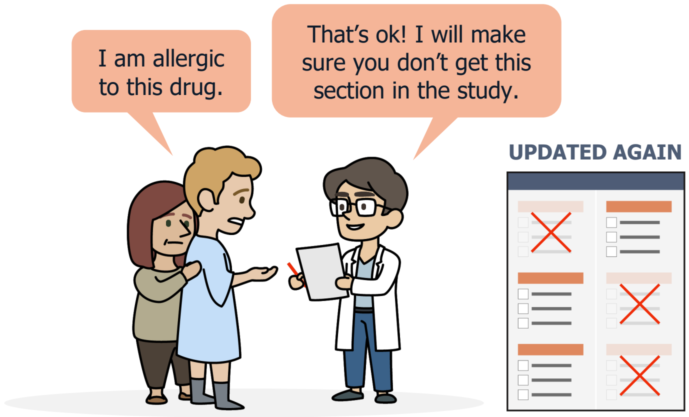

4 Your healthcare team will choose one or more sections left on your menu. Then a computer will choose one item from each of these sections.

These will be the items you will get in the study.

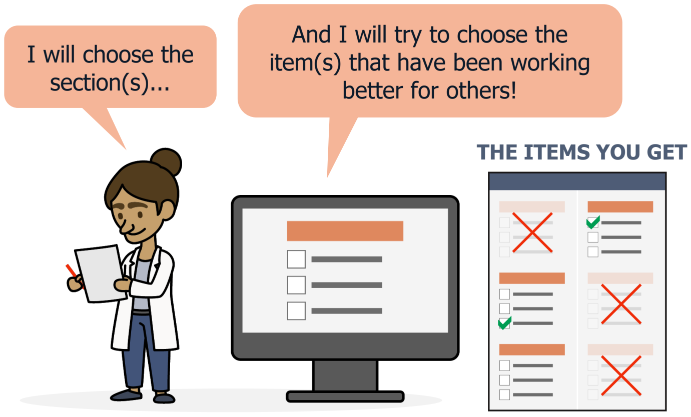

Your participation will not affect the quality of your care

You get to decide if you want to join the study. You can also leave the study at any time after joining.

- If you choose to join, you will be helping to improve care for future patients.
- If you choose not to join, you will still receive the best standard of care.

This document is confidential. Please do not share this document with others.

Your Menu

P For pneumonia   C For COVID-19

Antibiotics P C

Antibiotics are widely used in usual care to treat infections caused by bacteria. COVID-19 is caused by a virus but can lead to bacterial infections.

INTERVENTIONS

REMAP-CAP studies which combinations of antibiotics are most effective in treating pneumonia and/or COVID-19.

- ❑ Ceftriaxone + azithromycin
- ❑ Piperacillin-tazobactam + azithromycin
- ❑ Levofloxacin or moxifloxacin

HOW YOU GET IT

Through mouth, feeding tube, or a thin tube inserted into your vein (called an intravenous).

Azithromycin Duration P C

Azithromycin is an antibiotic that is widely used in usual care to reduce inflammation. Duration refers to the length of the azithromycin treatment.

INTERVENTIONS

REMAP-CAP studies what azithromycin duration is most effective in treating pneumonia and/or COVID-19.

- ❑ Azithromycin for 3 to 5 days
- ❑ Azithromycin for 14 days

HOW YOU GET IT

Through mouth, feeding tube, or a thin tube inserted into your vein (called an intravenous).

Antivirals P C

Antivirals are widely used in usual care to treat infections caused by some but not all viruses.

INTERVENTIONS

Oseltamivir is an antiviral. REMAP-CAP studies which oseltamivir option is most effective in treating pneumonia suspected to be caused by the influenza virus.

- ❑ Oseltamivir for 5 days
- ❑ Oseltamivir for 10 days
- ❑ No oseltamivir

HOW YOU GET IT

Through mouth or feeding tube.

Blood Thinners C

Blood thinners are widely used in usual care to prevent or reduce blood clots. COVID-19 can lead to blood clots and inflammation.

INTERVENTIONS

REMAP-CAP studies whether blood thinners are effective in treating COVID-19. It is not known how much blood thinners should be given in COVID-19 or for how long.

- ❑ Low dose
- ❑ Medium dose
- ❑ Full dose

HOW YOU GET IT

Through a thin tube inserted into your vein (called an intravenous) or injected just under the skin.

Corticosteroids P

Corticosteroids are widely used in usual care to reduce inflammation.

INTERVENTIONS

Hydrocortisone is a corticosteroid. REMAP-CAP studies which hydrocortisone option is most effective in treating pneumonia.

- ❑ Hydrocortisone for 7 days
- ❑ Hydrocortisone given only when in septic shock (i.e. severe infection)
- ❑ No corticosteroid

HOW YOU GET IT

Through a thin tube inserted into your vein (called an intravenous).

Statins C

Statins are widely used in usual care to reduce cholesterol (fats) in the blood, and may also reduce inflammation.

INTERVENTIONS

Simvastatin is a statin. REMAP-CAP studies whether simvastatin is effective in treating COVID-19.

- ❑ Simvastatin for up to 28 days
- ❑ No simvastatin

HOW YOU GET IT

Through mouth or feeding tube.

This document is confidential. Please do not share this document with others.

This is the end of Prototype 2.

Before moving on, imagine you received this resource as a patient in the ICU or as a substitute decision maker for a loved one in the ICU.

Please write down:

- 3 things you liked about this prototype
- 3 things you disliked about this prototype
- Overall feelings about this prototype

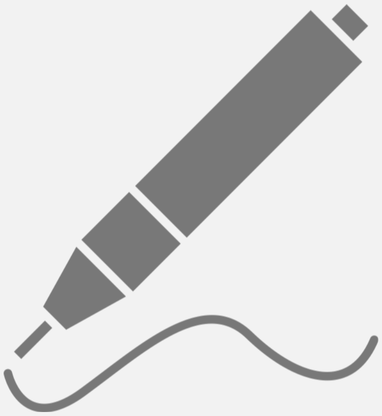

The following **2 pages** contain

Prototype 3

Please read both pages carefully.

This document is confidential. Please do not share this document with others.

What is REMAP-CAP?

REMAP-CAP is a research trial for patients with pneumonia or suspected or confirmed COVID-19. Participation in this trial is completely voluntary. You can opt out at any time even after joining.

Interventions are medications or treatments that have the potential to treat a condition.

Traditional trials
often test only **one intervention** at a time

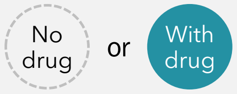

REMAP-CAP trial
tests **more than one intervention** at a time

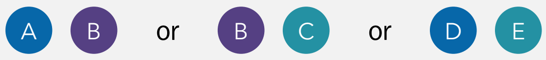

A simple way to think about REMAP-CAP:

All the interventions in the trial together is like a **deck of cards**

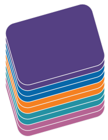

A single **intervention** is like a **card** in the deck

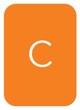

A **domain** (or category) that an intervention belongs to is like a **suit**

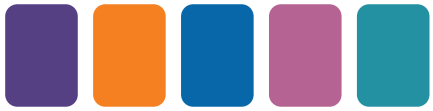

Your healthcare team has checked your medical history. They have **removed the suits that are not suitable for you** from your card deck on the back of this page. For example, you may be allergic to a drug.

Your card deck

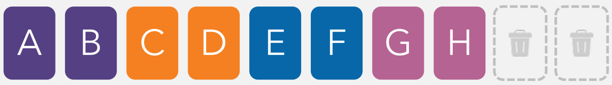

Discuss with your research coordinator if you are not comfortable with any of the suits left. They will also remove these suits from your card deck.

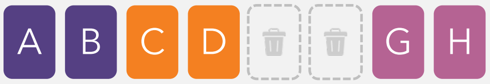

Your healthcare team will choose one or more suits left in your card deck.

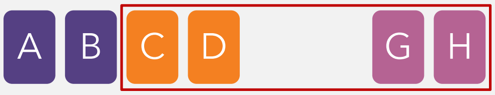

Then a computer will choose one card from each of those suits. These cards will be the interventions that you get in the trial. In this example, you will get interventions C and G.

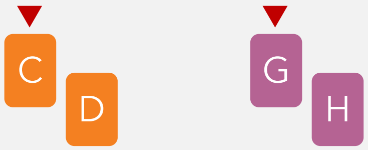

What is the difference between usual care and REMAP-CAP?

Standard care (usual care)
Standard care uses interventions (medications or treatments) that have been studied and are proven safe. Your doctor may give you one or more standard interventions even if you are not in the trial.

REMAP-CAP trial
REMAP-CAP tests many interventions used in standard care. But it may test the interventions in a new condition such as COVID-19. Or it may compare interventions to each other to see which is more effective.

What are the benefits and risks of joining the trial?

Benefits
There may or may not be direct benefits for you if you join the trial. Your data from the trial will help researchers improve care for future patients.

Risks
Each intervention has its own risks. Your research coordinator will review each intervention and its risks with you. You get to decide which interventions you are comfortable with.

Your card deck

DOMAINS	✓ INTERVENTIONS	NOTES
**Antibiotics**<br>*For pneumonia and COVID-19*<br>Antibiotics help to fight some types of infection caused by bacteria. Some viruses can also lead to bacterial infections.	☐ Ceftriaxone + azithromycin<br>☐ Piperacillin-tazobactam + azithromycin<br>☐ Levofloxacin or moxifloxacin	
**Azithromycin duration**<br>*For pneumonia and COVID-19*<br>Azithromycin is an antibiotic. Duration refers to how long you would receive azithromycin for.	☐ Azithromycin for 3 to 5 days<br>☐ Azithromycin for 14 days	
**Antivirals**<br>*For pneumonia and COVID-19*<br>Antivirals help to fight infections caused by viruses. COVID-19 is caused by a virus.	☐ No oseltamivir<br>☐ Oseltamivir for 5 days<br>☐ Oseltamivir for 10 days	
**Blood thinners**<br>*For COVID-19 only*<br>Blood thinners help to prevent or reduce blood clotting. It is not known how effective blood thinners are in treating COVID-19.	☐ Low dose blood thinner<br>☐ Intermediate dose blood thinner<br>☐ Continuation of full dose blood thinner	
**Corticosteroids**<br>*For pneumonia only*<br>Corticosteroids help to fight inflammation.	☐ No corticosteroid<br>☐ Fixed duration hydrocortisone for 7 days<br>☐ Hydrocortisone given only in septic shock	
**Statins**<br>*For COVID-19 only*<br>Statins help to reduce serum cholesterol and may reduce inflammation. It is not known how effective statins are in treating COVID-19.	☐ No simvastatin<br>☐ Simvastatin for up to 28 days	

This document is confidential. Please do not share this document with others.

This is the end of Prototype 3.

Before moving on, imagine you received this resource as a patient in the ICU or as a substitute decision maker for a loved one in the ICU.

Please write down:
- 3 things you liked about this prototype
- 3 things you disliked about this prototype
- Overall feelings about this prototype

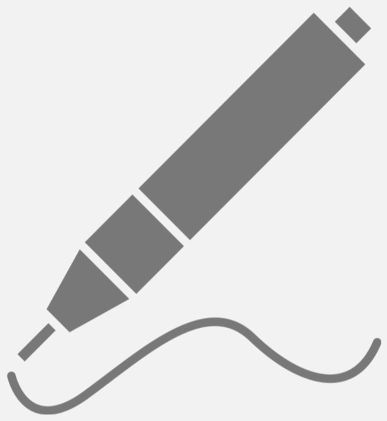

This document is confidential. Please do not share this document with others.

The following **14 pages** contain the

Consent form

KEEP IN MIND

- Often the research coordinator (RC) will only verbally summarize the main points in this consent form for the patient or substitute decision maker (SDM). It is not likely for the RC to go over the form word for word with the patient or SDM.
- If the patient or SDM would like to read through the consent form in detail before making their decision, the RC will give them a copy to review.

Please read all pages carefully.

Letter of Information and Consent to Participate in a Research Study
[Insert site name]

COVID-19: Pandemic infection is either suspected or proven

**Title**	Randomized, Embedded, Multifactorial Adaptive Platform trial for Community-Acquired Pneumonia
**Short Title**	REMAP-CAP
**Project Funder**	Canadian Institutes of Health Research
**Coordinating Principal Investigator/ Principal Investigator**	Dr.John Marshall/ Dr. Srinivas Murthy[Principal Investigator]
**Co-Investigator(s)** *(if required by institution)*	[Co-Investigator(s)]
**Emergency Contact Information**	[Phone number]

(The description of the domain + associated risks are outlined in an appendix that will be/is provided alongside the main consent form.)

This consent form is intended for the participant who is eligible to take part in this study. However, if the participant is incapable of providing consent due to the severity of his/her illness, the consent of a relative or other authorized representative/substitute decision maker will be sought. As a Substitute Decision Maker (SDM), you are being asked to provide informed consent on behalf of a person who is unable to provide consent for him/herself. If the participant gains the capacity to consent for him/herself, your consent for them will end and we will seek his/her consent directly.

Note: the terms "you" or "your" refer to the person being asked to participate in this research. The remainder of this document should help you in this decision.

This consent form provides you with information to help you make an informed choice. Please read this document carefully and ask any questions you may have. All of your questions should be answered to your satisfaction before you decide whether to participate in this research study.

Please take your time in making your decision. You may find it helpful to discuss it with your friends and family.

Taking part in this study is voluntary. Deciding not to take part or deciding to leave the study will not result in any penalty or affect current or future health care of you or your family.

There are no conflicts of interest to declare related to this study.

Part 1

1 Introduction

You are being invited to take part in this research project. This is because you have an acute illness due to suspected or proven pandemic infection affecting the lungs which may be caused by a new Coronavirus. The disease is called COVID-19.

This document is confidential. Please do not share this document with others.

Most patients with suspected or proven infection due to COVID-19 who are being treated in the hospital will receive many different study drug interventions some of which are part of standard care while some drug interventions were originally approved by Health Canada for different medical conditions. These study drugs may potentially have beneficial effects for COVID-19 infection, acting on both the infection and its effects on the body. For this research project:

- Several drug interventions may be tested, at the same time, in the same patient.
- We will tell you if you are not eligible for all options that are tested in the study.
- You can choose the parts of the study you participate in.
- Because COVID-19 is a new disease, doctors don't already know which interventions work and the side effects of these options.
- The study looks at its results as it goes and uses the results so that new participants in the study have a better chance of getting better interventions.
- The different study drug options are tested to tell which are the best. The study only tests options where it is not known which interventions are best.

2 About this Information Sheet and Consent Form

This Participant Information Sheet/Consent Form tells you about the research project. It explains the interventions involved and how the study drugs you will receive will be determined. Knowing what is involved will help you decide if you want to take part in the research.
Please read this information carefully. Ask questions about anything that you don't understand or want to know more about. Before deciding whether or not to take part, you might want to talk about it with a relative, friend or your doctor.

Participation in this research is voluntary. If you don't wish to take part, you do not have to. You will receive the best possible care whether or not you take part.

If you decide you want to take part in the research project, you will be asked to sign the consent section. By signing it you are telling us that you:

- Understand what you have read;
- Agree to taking part in the research project;
- Agree to having the tests and interventions that are described, so long as the doctor thinks they are appropriate;
- Agree to the use of your personal and health information as described below.

You will be given a copy of this Participant Information and Consent Form to keep.

3 What is the purpose of this research?

The purpose of this research is to improve survival and recovery for patients with acute illness due to suspected or proven COVID-19 infection or pneumonia.

The doctors and the team associated with this research project are dedicated to giving you the best possible care based on the results from the different interventions.

Patients with pneumonia receive different drug interventions in order to determine what works best. This study will determine what the best options are, and how to use them effectively.

The medications listed under Antibiotics, azithromycin, and the antiviral oseltamivir are already available and used routinely to treat patients with pneumonia.

This study addresses seven questions regarding potential treatment of COVID-19 infection or pneumonia:

COVID-19 use of immune modulation

Use of Vitamin C

Use of antibiotics

Duration of azithromycin treatment

Use of antiviral medication oseltamivir

COVID-19 use of antiplatelet

COVID-19 use of statin therapy

4 What does participation in this research involve?

REMAP-CAP is a 'Platform Trial' that studies several different interventions at the same time, and assigns participants to arms using a process called "***response-adaptive randomization***". Randomization means that you are put into a group by chance (like flipping a coin). There is no way to predict which group you will be assigned to. Neither you nor the study team can choose what group you will be in. Response-adaptive randomization or RAR involves frequent analysis of the study results as the trial progresses; if one particular arm appears to be doing better, the randomization process is modified so that subsequent participants are more likely to get the arm that is performing better. As the trial continues, participants are more likely to be enrolled in each of the study arms that seem to be doing better.

During the study, your doctor knows what study drugs you are getting at all times, and that the research is appropriate and suitable for you. This means the doctor can change your study drugs if he or she feels that is appropriate. He or she will not know how the trial is trending until recruitment has been completed, the stopping point being based a pre-determined threshold for concluding benefit, harm, or no effect.

There are no costs to you for participating in this research project. You will not be paid for your participation in this project.

5 What do you have to do?

In hospital
Members of the research team will collect information from your medical record and enter this information in a database so it can be analysed. This information will be only items that are routinely collected during your hospitalization, such as what study drugs are provided, lab results, and how long you are in the hospital.

Following discharge from hospital
You will be contacted by a trained research co-ordinator by telephone approximately 3 months after you started in the study. This phone call is to determine how well you are recovering. It should take no longer than 5 minutes.

At approximately 6 months after you start in the study, you will be contacted for information about your recovery. A trained research coordinator will ask about your quality of life (i.e. if you are able to walk, look after yourself and generally how you are doing). It is expected that this phone call will take no more than 10 minutes. We will ask you to supply contact phone numbers at the start of the study. The questions for the phone interview can be mailed to you a few weeks before the phone call to save time during the call.

6 Do I have to take part in this research project?

INCLUDE DOMAINS AND INTERVENTIONS THAT YOUR SITE HAS SELECTED.

This document is confidential. Please do not share this document with others.

Participation in research is voluntary. If you do not wish for to take part, you do not have to. If you decide to take part and later change your mind, you are free to withdraw from the project at any time.
REMAP-CAP is looking at areas where the normal approach to treatment varies amongst doctors – the initial choice of antibiotic, whether to prolong treatment with the antibiotic azithromycin because of a possible effect on bolstering the body's immune system, and whether or not to treat with an anti-viral medication Oseltamivir. REMAP-CAP is also looking at areas where certain drugs may have beneficial effect on patients with suspected or proven COVID-19 infection – the immune modulators interferon-beta1a and anakinra, Vitamin C, antiplatelets, and statins.

To participate in the trial, you must agree to be randomized to at least one of these domains. At the same time, you can also decline to participate in any of the arms under the domain (provided you are enrolled in at least one arm). If you decline participation in any of the study arms, you will receive usual care as guided by your doctor. This is likely to include one of the study arms, however that decision will reflect your doctor's preference, and because it is outside the study protocol, the details will not contribute to the study conclusions.
If you do decide to take part, you will be given a Participant Information and Consent Form to sign and you will be given a copy to keep. You will be told, in a timely manner, about new information that may be relevant to your willingness to stay in this research project.

If it turns out that you don't have COVID-19 infection, you will continue to receive the interventions that you were assigned into. Your doctor has considered the safety of these interventions even if your test for COVID-19 is negative, before you were enrolled into the study.

Since the study drugs have not undergone extensive investigation of the potential side effects on the unborn child, if you are of childbearing potential, we will request for a pregnancy test prior to enrolment. The result will help in the selection of interventions that will be offered to you.

Your decision to take part, NOT take part, or take part and then withdraw, will not affect you or your family's routine treatment, your relationship with those treating you, or your relationship with [Institution].

7 What are the alternatives to participation?

If you decide NOT to participate in this research project, your doctor will choose to treat you based on his/her best clinical judgment according to standard care (but will not have access to the results of the study).

8 What are the possible benefits of taking part?

We cannot promise that you will receive any benefits from this research; however you will contribute to improving the care of patients with pneumonia.

9 Can I have other treatments during this research project?

Participation in this research project will not affect other medications or treatments you have been taking. It is important to tell your study doctor and the study staff about any treatments or medications you may be taking, including over-the-counter medications, vitamins or herbal remedies, acupuncture or other alternative treatments. You should also tell your study doctor about any changes to the medications you remain on or during your participation in the research project. Participation in REMAP-CAP will also not affect your ability to take part in other studies during the course of your illness.

10 Who has reviewed the research project?

REMAP-CAP has undergone two levels of review. First the project itself has been evaluated for its scientific validity and importance by the Canadian Institutes of Health Research, and by funding bodies in Australia, New Zealand, and the European Union. In addition, all research in Canada

This document is confidential. Please do not share this document with others.

involving humans is reviewed by an independent group of people called a Research Ethics Board (REB). The ethical aspects of this research project have been reviewed by the Unity Health Toronto Research Ethics Board.
Any person with questions about their rights as a research participant should contact the REB at 416-864-6060 ext. 2557 during business hours (9:00 am – 5:00 pm).

11 Can I withdraw from this research project?

If you decide to withdraw from the project, please notify a member of the research team. This notice will allow a member of the research team to discuss any health risks or special requirements linked to withdrawal. Data that has already been entered into the database will remain there to inform the study question; we would also request that we continue to collect data on your health status at day 90 and up to 180 days from the day you entered the study. However, you have the option to withdraw or request removal of your data upon withdrawal from the study. You can discuss this with the research team.

12 What will happen to my information?

By signing the consent form, you consent to the study doctor and relevant research staff to collect your personal information for the research project. Any information obtained in connection with this research project that can identify you will be removed - this is called 'de-identification', and all data sent to the central data centre will identify you by a study number. This number can only be linked to your identification through a Master Linking Log which will be maintained under lock and key at the hospital where you are being cared for. Information relating to the study will be kept for 25 years, in accordance with Canadian regulations. Information about participation in this research project may be recorded in your health records.
By signing the Consent Form, you authorise the release of, or access to, this confidential information by relevant study personnel. Representatives of Clinical Trials Ontario, a not-for-profit organization, may see study data (information that is collected about you for the research project) that is sent to the research ethics board for this study. Identifiable information will not be used. Authorized representatives of the following organizations may look at your original (identifiable) medical/clinical study records at the site where these records are held, to check that the information collected for the study is correct and follows proper laws and guidelines.

- The research ethics board who oversees the ethical conduct of this study in Ontario
- [Insert research site name], to oversee the conduct of research at this location

All research information will be collected from your medical record by the research staff at [hospital name]. This study information will be kept in a locked filing cabinet, or a password protected computer in the secure research office at [hospital name], which is only accessible to the research staff. Your de-identified research information will be sent to the coordinating centre in Melbourne, Australia and to the Intensive Care National Audit and Research Centre (ICNARC) in the UK. ICNARC will manage the study data on behalf of the coordinating centre in Australia and will analyse data from the other study sites. Information about your name and contact details, for the purposes of follow-up, will be kept at [hospital name]. This contact information will not be stored together with any other research information, and will not be sent to Australia or UK. The research study results will be held securely throughout, and no identifiable information will be sent outside of [hospital name].

If participating in the Vitamin C domain, the de-identified data collected will be shared with investigators of another Vitamin C study looking at the same outcome in patients with suspected or proven COVID-19. These investigators are part of the REMAP-CAP group who will be performing data analysis from pooled data.

The study personnel will make every effort to keep your personal health information private and confidential in accordance with all applicable privacy legislation, including the Personal Health Information Protection Act (PHIPA) of Ontario. In accordance with relevant Canadian privacy and other relevant laws, you have the right to request access to any of your information collected and

stored by the study team. You also have the right to correct any information with which you disagree. Please contact the study team member named at the end of this document if you would like to access your information.
Any information transferred to the coordinating centre in Australia and ICNARC will be in compliance with all relevant Canadian privacy laws. By signing this consent form, you are consenting to the disclosure of your de-identified information to organizations located outside of Canada. Any information sent outside of Canadian borders may increase the risk of disclosure of information because the laws in those countries dealing with protection of information may not be as strict as in Canada. Even though the likelihood that someone may identify you from the study data is very small, it can never be completely eliminated.

COVID-19 Information

If you are tested for COVID-19 before or at any time during this study, the study sponsor may want to know the results of this testing, whether the result is positive or negative. Since we do not fully understand how COVID-19 affects different people, it may be a meaningful factor to consider in this study.

Please be aware that if you have a serious side effect or other medical issue during the study, your COVID-19 status (if known) may be included in a report sent to the sponsor and/or regulatory agencies for safety reasons.

Adding Information into Your Medical Record

Your participation in this study will be recorded in your [hospital name] medical record. If you participate in this study, throughout the study we will collect medical information from your chart. We will be documenting lab results, medications, vital signs, documentation of consent discussion, consent form, study drug dosing, and results of tests done for study purposes.

[Hospital name] may share patient information stored on its electronic medical records system with other hospitals and health care providers in Ontario so they can access the information if it is needed for your clinical care. Any of these people may see that you were in this study and the study data listed above when they access your medical record for clinical purposes.

13 Compensation for Injury

If you suffer any injuries or complications as a result of this research project, you should contact the study team as soon as possible and you will be assisted in accessing appropriate medical treatment.

By signing this form you do not give up any of your legal rights against the study doctor, sponsor or involved institutions for compensation, nor does this form relieve the study doctor, sponsor or their agents of their legal and professional responsibilities.

14 Further information and who to contact

The person you may need to contact will depend on the information you are seeking.

If you want any further information concerning this project, or if you have any medical problems which may be related to your involvement in the project (for example, any side effects or feelings of distress), you can contact the principal study doctor [name] at [Contact phone number].

For matters relating to your rights as a research participant, you may contact the Research Ethics Board (REB):

REB contact person

Name	*Dr. David Mazer*
Position	*Unity Health Toronto Research Ethics Board Chair*
Telephone	416-864-6060 ext. 2557

This document is confidential. Please do not share this document with others.

Part 2

15 How is this study being paid for?

This study is being conducted in Australia, New Zealand, Europe and Canada. In Canada, the research is being run at St. Michael's Hospital on behalf of a large group of ICU doctors, nurses, and other health professionals known as the Canadian Critical Care Trials Group.

The cost of some interventions used for COVID-19 may be covered by pharmaceutical companies that make these products. These pharmaceutical companies have no involvement in the design, analysis or reporting of results from the trial.

This research has been funded by the Canadian Institutes of Health Research (CIHR).

No money is paid directly to individual researchers or doctors.

16 Who is organising the research?

This research project is being conducted by St. Michael's Hospital.

[Name of institution] will receive a payment from St. Michael's Hospital for undertaking this research project.

No member of the research team will receive a personal financial benefit from your involvement in this research project (other than their ordinary wages).

17 Other relevant information about the research project

We plan to conduct this research study at approximately 30 hospitals in Australia and New Zealand, 50 hospitals in Europe, and 40 hospitals in Canada. We expect about 7,100 patients to participate over the first 4 years, with anywhere between 10-50 patients at each Canadian site over that time.

A description of this clinical trial will be available on http://www.clinicaltrials.gov. This website will not include information that can identify you. You can search this website at any time. The registration number for this study is NCT02735707.

18 What if new information arises during this research project?

Sometimes during the course of a research project, new information becomes available about the treatment being studied. If this happens, the research team, in combination with a group of independent doctors and research experts (known as the Data Safety Monitoring Board) will decide how best to communicate this new information.

19 Could this research project be stopped unexpectedly?

This research project may be stopped unexpectedly for a variety of reasons. These may include reasons such as:

- Unacceptable side effects
- The drug/intervention being shown not to be effective
- The drug/intervention being shown to work and not need further testing

If you are removed from this study, the study doctor will discuss the reasons with you and plans will be made for your continued care outside of the study.

20 What happens when the research project ends?

This document is confidential. Please do not share this document with others.

It is anticipated that the results of this research project will be published and or presented in a variety of forums. In any publication and/or presentation, information will be provided in such a way that you cannot be identified. The study website www.remapcap.org will provide updates on the study's progress and publications.

This document is confidential. Please do not share this document with others.

Consent Form – Participant Statement of Consent
COVID-19: Pandemic infection is either suspected or proven

**Title**	Randomized, Embedded, Multifactorial Adaptive Platform trial for Community-Acquired Pneumonia
**Short Title**	REMAP-CAP
**Project Funder**	Canadian Institutes of Health Research
**Coordinating Principal Investigator/ Principal Investigator**	Dr. John Marshall/Dr.Srinivas Murthy/ [Principal Investigator]
**Co-Investigator(s)** *(if required by institution)*	[Co-Investigator(s)]
**Emergency Contact Information**	[Phone number]

The research study has been explained to me, and my questions have been answered to my satisfaction.

I have been informed of the alternatives to participation in this study. I have the right not to participate and the right to withdraw without affecting the quality of medical care at (Hospital's Name) for me and for other members of my family. As well, the potential harms and benefits (if any) of participating in this research study have been explained to me.

I have been told that I have not waived my legal rights nor released the investigator, sponsor, or involved institutions from their legal and professional responsibilities.

I know that I may ask now, or in the future, any questions I have about the study.

I have been told that records relating to me and my care will be kept confidential and that no information will be disclosed without my permission unless required by law.

I have been given sufficient time to read the above information.

The appendices were provided to me with the consent form, I have read the information, and had a chance to ask questions. □ Yes □ No

I freely agree to participate in the following arms of this research project:

			For RC:		
**Use of COVID-19 immune modulation**	☐ **Yes**	☐ **No**	☐ ***N/A***		
**Use of Vitamin C**	☐ **Yes**	☐ **No**	☐ ***N/A***	☐***ICU***	☐***Ward***
**Choice of antibiotic**	☐ **Yes**	☐ **No**	☐ ***N/A***		
**Duration of azithromycin treatment**	☐ **Yes**	☐ **No**	☐ ***N/A***		
**Use of influenza antiviral medications**	☐ **Yes**	☐ **No**	☐ ***N/A***		
**Use of COVID-19 antiplatelets**	☐ **Yes**	☐ **No**	☐ ***N/A***	☐***ICU***	☐***Ward***
**Use of COVID-19 statins**	☐ **Yes**	☐ **No**	☐ ***N/A***	☐***ICU***	☐***Ward***

I consent to participate in this study. I will be given a copy of the signed and dated consent form.

____________________	____________________	____________________
Name of Participant (print)	Signature of Participant	Date/Time (24h clock)

I have explained to the above-named participant the nature and purpose, the potential benefits, and possible risks associated with participation in this research study. All questions that have been raised about the research have been answered.

____________________	____________________	____________________
Name & Position of Person Obtaining Consent (print)	Signature of Person Obtaining Consent	Date/Time (24h clock)

This document is confidential. Please do not share this document with others.

Consent Form – Substitute Decision Maker (SDM)
COVID-19: Pandemic infection is either suspected or proven

**Title**	Randomized, Embedded, Multifactorial Adaptive Platform trial for Community-Acquired Pneumonia
**Short Title**	REMAP-CAP
**Project Funder**	Canadian Institutes of Health Research
**Coordinating Principal Investigator/ Principal Investigator**	Dr. John Marshall/Dr.Srinivas Murthy/ [Principal Investigator]
**Co-Investigator(s)** *(if required by institution)*	[Co-Investigator(s)]
**Emergency Contact Information**	[Phone number]

Consent Agreement

I am the Substitute Decision Maker for *[Participant's Name]* (the Participant).

I have read the Participant Information Sheet or someone has read it to me in a language that I understand.

I understand the purposes, procedures and risks of the research described in the project.

I have had an opportunity to ask questions and I am satisfied with the answers I have received.

I believe that participation in this study is not contrary to the participant's best interests/preferences and values and social wellbeing.

I acknowledge that the participant does not give up any of their legal rights by participating.

I freely agree to the participant's involvement in the following arms of this research project:

			For RC:		
**Use of COVID-19 immune modulation**	☐ **Yes**	☐ **No**	☐ ***N/A***		
**Use of Vitamin C**	☐ **Yes**	☐ **No**	☐ ***N/A***	☐ ***ICU***	☐ ***Ward***
**Choice of antibiotic**	☐ **Yes**	☐ **No**	☐ ***N/A***		
**Duration of azithromycin treatment**	☐ **Yes**	☐ **No**	☐ ***N/A***		
**Use of influenza antiviral medications**	☐ **Yes**	☐ **No**	☐ ***N/A***		
**Use of COVID-19 antiplatelets**	☐ **Yes**	☐ **No**	☐ ***N/A***	☐ ***ICU***	☐ ***Ward***
**Use of COVID-19 statins**	☐ **Yes**	☐ **No**	☐ ***N/A***	☐ ***ICU***	☐ ***Ward***

As described I understand that the participant is free to withdraw from this study at any time without affecting future health care for them or their family.
I am aware of my responsibilities as the substitute decision maker for the participant and I understand that I will be assisting the participant in meeting their responsibilities while they are participating in this study.

This document is confidential. Please do not share this document with others.

If I, on behalf of the participant, decide to discontinue the treatment, a member of the research team may request permission to obtain access to the participant's medical records for collection of follow-up information for the purposes of research and analysis.

I understand that I will be given a signed copy of this document to keep on behalf of the participant.

The appendices were provided to me with the consent form, I have read the information, and had a chance to ask questions. □ Yes □ No

Declaration by the Substitute Decision Maker who has read the information

Name of Participant (please print)

____

Name of Person providing consent (please print)

____

Relationship of Person providing consent to Participant _________________________

Signature of Person providing consent ________________________ Date _______

Declaration by Participant/Substitute Decision Maker unable to read the information and consent form*

Witness to the informed consent process
The informed consent form was accurately explained to, and apparently understood by, the participant/substitute decision maker, AND
Informed consent freely given by the participant/substitute decision maker
Name (please print) ____
Signature _______________________ Date ______________________
* Witness is not to be the investigator, a member of the study team or their delegate. In the event that an interpreter is used, the interpreter may not act as a witness to the consent process. Witness must be 18 years or older.

Declaration by Research Team

I have given a verbal explanation of the research project, its procedures and risks and I believe that the participant/substitute decision maker has understood that explanation.

Name of Researcher (please print) ___________________________________

Signature ______________________ Date ______________________

Note: All parties signing the consent section must date their own signature

Form for Withdrawal of Participation – Participant/Substitute Decision Maker
COVID-19: Pandemic infection is either suspected or proven

**Title**	Randomized, Embedded, Multifactorial Adaptive Platform trial for Community-Acquired Pneumonia
**Short Title**	REMAP-CAP
**Project Funder**	Canadian Institutes of Health Research
**Coordinating Principal Investigator/ Principal Investigator**	Dr. John Marshall/Dr.Srinivas Murthy/ [Principal Investigator]
**Co-Investigator(s)** *(if required by institution)*	[Co-Investigator(s)]
**Emergency Contact Information**	[Phone number]

Declaration by Participant/Substitute Decision Maker

I wish to withdraw/withdraw the participant from taking part in the above research project:

			For RC:
**Use of COVID-19 immune modulation**	☐ **Yes**	☐ **No**	☐ ***N/A***
**Use of Vitamin C**	☐ **Yes**	☐ **No**	☐ ***N/A***
**Choice of antibiotic**	☐ **Yes**	☐ **No**	☐ ***N/A***
**Duration of azithromycin treatment**	☐ **Yes**	☐ **No**	☐ ***N/A***
**Use of influenza antiviral medications**	☐ **Yes**	☐ **No**	☐ ***N/A***
**Use of COVID-19 antiplatelets**	☐ **Yes**	☐ **No**	☐ ***N/A***
**Use of COVID-19 statins**	☐ **Yes**	☐ **No**	☐ ***N/A***

I understand that such withdrawal will not affect my/participant's routine treatment, relationship with those treating me/them or my/their relationship with [Institution].

Name of Participant (please print)

____

Name of Person providing consent (please print)

____

Relationship of Person providing consent to Participant _________________________

Signature of Person providing consent ________________________ Date _______

Name of Study Doctor(please print) ____________________________________

Signature ______________________ Date ______________________

Note: All parties signing the consent section must date their own signature.

This document is confidential. Please do not share this document with others.

Study Title: Randomized, Embedded, Multifactorial Adaptive Platform trial for Community-Acquired Pneumonia (REMAP-CAP)

COVID-19: Pandemic infection is either suspected or proven

Statement of Consent (Participant capacity regained)

By signing this consent form, I acknowledge that:

- I understand that permission was given for me to participate in this study by my Substitute Decision Maker while I was too sick to make my own decisions.
- The research study has now been explained to me, and my questions have been answered to my satisfaction.
- I have been informed of the alternatives to participation in this study.
- I know that I have the right not to continue participating in this study and the right to withdraw without affecting the quality of medical care at [Hospital name] for me and for other members of my family.
- The potential harms and benefits (if any) of participating in this research study have been explained to me.
- I have been told that I have not waived my legal rights nor released the investigator, sponsor, or involved institutions from their legal and professional responsibilities.
- I know that I may ask now, or in the future, any questions I have about the study.
- I have been told that records relating to me and my care will be kept confidential and that no personal information will be disclosed without my permission unless required by law.
- I have been given sufficient time to read all pages of this document.
- I will be given a copy of this consent form.
- The appendices were provided to me with the consent form, I have read the information, and had a chance to ask questions. ☐ Yes ☐ No

At this time, I am now able to make my own decisions and (check and initial as decided):

	**YES, I agree** to allow my collected study data to remain part of the study.
	**NO, I do not consent** to allow my collected study data to remain part of the study

I freely agree to participate in the following arms:

			For RC:
**Use of COVID-19 immune modulation**	☐ **Yes**	☐ **No**	☐ ***N/A***
**Use of Vitamin C**	☐ **Yes**	☐ **No**	☐ ***N/A***
**Choice of antibiotic**	☐ **Yes**	☐ **No**	☐ ***N/A***
**Duration of azithromycin treatment**	☐ **Yes**	☐ **No**	☐ ***N/A***
**Use of influenza antiviral medications**	☐ **Yes**	☐ **No**	☐ ***N/A***
**Use of COVID-19 antiplatelets**	☐ **Yes**	☐ **No**	☐ ***N/A***
**Use of COVID-19 statins**	☐ **Yes**	☐ **No**	☐ ***N/A***

____________________	____________________	____________________
Participant Name (print)	Participant Signature	Date and Time

I have explained to the above-named participant the nature and purpose, the potential benefits, and possible risks associated with participation in this research study. All questions that have been raised about the research have been answered.

____________________	____________________	____________________
Name & Position of Person Obtaining Consent (print)	Signature of Person Obtaining Consent	Date and Time

The following **7 pages** contain the

Domain appendices

KEEP IN MIND

- On top of the consent form, the research coordinator (RC) will share with the patient or substitute decision maker (SDM) important information about each domain that the patient may receive in the trial.
- Each domain has its own appendix. **A patient may receive a few or many domain appendices depending on their unique health history and situation.**
- The RC will only verbally summarize the main points in these appendices during the consent process. It is not likely for the RC to go over each appendix word for word with the patient or SDM.
- If the patient or SDM would like to read through each appendix in detail before making their decision, the RC will give them a copy to review.

Please read all appendices if you have time.
If not, please read at least ONE appendix.

REMAP-CAP
Randomized, Embedded, Multifactorial, Adaptive Platform trial for Community-Acquired Pneumonia

Choice of antibiotic. All patients with pneumonia are given antibiotics, but the choice of antibiotics varies amongst doctors. This project compares 3 antibiotics that are commonly used in Canada:

Ceftriaxone + azithromycin
Piperacillin-tazobactam + azithromycin
Moxifloxacin/Levofloxacin

The doctors in this ICU have chosen to have these options available in the study because all of these options are known or believed to be safe and effective. If the participant is not in the study, it is very likely that the doctors would have treated them with one of these options. However, it is not known which option is best. The choice of antibiotic evaluates different types of "standard care". The doctors in this ICU will be asked to consider whether the above antibiotics are appropriate for your individual medical presentation.

You will not receive an antibiotic to which you have an allergy.

What are the possible risks and disadvantages of taking part?

The drugs included in the antibiotic domains are all part of standard care and commonly provided to patients with pneumonia. They often cause side-effects. The risks from side effects in this domain will be similar whether you participate in the study or not. Your doctor will know what drug you are receiving at all times, and will be looking out for side effects, and your doctor will also have to agree to you participating in this study. As the drugs in this domain are all commonly used, the doctors and nurses who will be caring for you in the ICU are trained to recognize the associated risks. If side effects occur, and the doctor thinks it is best to stop that drug that is what will happen.

What are the reproductive risks?

If you could be pregnant or are breast feeding, the study will only give options in the antibiotic domain that are recommended for use in these situations.

This document is confidential. Please do not share this document with others.

REMAP-CAP
Randomized, Embedded, Multifactorial, Adaptive Platform trial for Community-Acquired Pneumonia

Duration of azithromycin treatment. Azithromycin is an antibiotic that also has anti-inflammatory actions. Most doctors who give azithromycin to patients stop it after a few days when its antibiotic effects are done. When it is stopped early the patient does not continue to benefit from its anti-inflammatory effect. In this research project, stopping azithromycin after a few days will be compared with continuing it for up to 14 days. The longer course of azithromycin is not a usual treatment, so this evaluation is a "new treatment".

Azithromycin for 3-5 days
Azithromycin for 14 days

What are the possible risks and disadvantages of taking part?

Azithromycin is part of standard care and commonly provided to patients with pneumonia. They often cause side-effects. The risks from side effects in this domain will be similar whether you participate in the study or not. Your doctor will know what drug you are receiving at all times, and will be looking out for side effects, and your doctor will also have to agree to you participating in this study. As the drug in this domain is commonly used, the doctors and nurses who will be caring for you in the ICU are trained to recognize the associated risks. If side effects occur, and the doctor thinks it is best to stop that drug that is what will happen.

What are the reproductive risks?

If you could be pregnant or are breast feeding, azithromycin is generally safe to use in these situations.

REMAP-CAP
Randomized, Embedded, Multifactorial, Adaptive Platform trial for Community-Acquired Pneumonia

Use of antiviral medication. When a patient has pneumonia caused by an influenza some doctors will prescribe a drug called Oseltamivir, an antiviral medication. Some doctors do not routinely use Oseltamivir, and those who do may prescribe it for different lengths of time. At this site, this project evaluates:

No Oseltamivir
Oseltamivir for five days
Oseltamivir for ten days

The doctors in this ICU have selected these options because they do not know which of them is best, but believe that all of these options are safe and effective. Therefore, these options are different types of "standard care". The participant will only receive these interventions if they have pneumonia that is believed or known to be caused by Influenza.

What are the possible risks and disadvantages of taking part?

Oseltamivir is part of standard care and commonly provided to patients with pneumonia. They often cause side-effects. The risks from side effects in this domain will be similar whether you participate in the study or not. Your doctor will know what drug you are receiving at all times, and will be looking out for side effects, and your doctor will also have to agree to you participating in this study. As the drug in this domain is commonly used, the doctors and nurses who will be caring for you in the ICU are trained to recognize the associated risks. If side effects occur, and the doctor thinks it is best to stop that drug that is what will happen.

What are the reproductive risks?

If you could be pregnant or are breast feeding, oseltamivir is generally safe to use in these situations.

This document is confidential. Please do not share this document with others.

REMAP-CAP
Randomized, Embedded, Multifactorial, Adaptive Platform trial for Community-Acquired Pneumonia

COVID-19 use of blood thinners. Early reports indicate that COVID-19 disease may cause inflammation and clots to form and this inflammation and these clots can cause serious harm. It is common practice to give some form of blood thinner to hospitalized patients to prevent the formation of blood clots. Patients with COVID-19 admitted to hospital are at high risk of clotting complications in spite of the low dose blood thinner given to prevent the formation of clots. Patients at highest risk are those with severe disease in the ICU. Because these clotting complications are preventable cause of deaths for patients with COVID-19, more intensive anti-clot prevention strategies or prophylaxis are being investigated to improve patient outcomes. The two most important strategies are the use of blood thinners and antiplatelets. At this site, this project evaluates:

Conventional low dose clot prevention (thromboprophylaxis)
Intermediate dose clot prevention (thromboprophylaxis)
Continuation of therapeutic dose anticoagulation

The low molecular weight heparins (LMWH) - Enoxaparin, Dalteparin, Tinzaparin - and Unfractionated Heparin (UFH) are the study drugs that will be used in this domain. The doses that you will be given will depend on what group you are assigned into.

UFH, if used, will be given through the vein and monitored according to local hospital policy, with guidelines used for the treatment of venous thromboembolism (VTE) or blood clots. LMWH is administered under the skin and monitored according to local hospital policy, practice and guidelines that pertain to prevention of blood clots.

No additional tests, examinations or samples of blood are required for this research study. If you are assigned to the therapeutic anticoagulation arm, your blood will be monitored and the dose of the UFH adjusted based on these results. The blood draw will be part of the clinical blood draw done daily while in the ICU. When the study is completed, whether to continue the anticoagulation will be at the discretion of your physician.

The allocated study drugs will be given until the end of study day 14 or until hospital discharge, whichever occurs first. After 14 days or hospital discharge, decisions regarding the use of blood thinners are at the discretion of your doctor.

The use of these doses of blood thinners in patients with severe COVID-19 is considered investigational. Health Canada has reviewed and authorized their use in this study.

What are the possible risks and disadvantages of taking part?

- Bleeding or oozing from the surgical wound – 4.9%
- Bleeding in the brain – 1%
- Bleeding in the stomach – 5.2%
- Heparin induced thrombocytopenia - a blood condition associated with low platelets (cells responsible for clotting) and blood clots - <1%
- Changes in the results of blood tests done to check how your liver is working – 6% (transient)
- Allergic reactions - rare

What are the reproductive risks?

This document is confidential. Please do not share this document with others.

If you are a female of childbearing potential, your physician will decide if participation in this domain is in your best interest and whether a pregnancy test is required before enrolment.

1) Dalteparin
The doses that will be used in this study does not appear to be associated with significant reproductive risks. In women with prosthetic heart valves, there have been reports of clot in the prosthetic valves while receiving the LMWHs for clot prevention. Available data have not reported a clear association with dalteparin and adverse effects on the fetus.

2) Enoxaparin
This LMWH should not be used in pregnant women unless the benefits outweigh the risks. There have been reports of congenital abnormalities in infants born to women who received LMWH during pregnancy.

3) Tinzaparin
Tinzaparin does not cross the placenta and can be used during all trimesters of pregnancy. Published data indicate that tinzaparin does not cause fetal malformation.

4) Unfractionated Heparin
Heparin use during pregnancy may be associated with perinatal death and prematurity. You must not become pregnant or father a baby while taking unfractionated heparin. The study doctor will discuss family planning with you to ensure that you do not become pregnant or father a baby during the study.

This document is confidential. Please do not share this document with others.

Use of corticosteroids (hydrocortisone). Hydrocortisone is an anti-inflammatory medication. Some doctors believe it helps reduce inflammation in the lungs and elsewhere in the body, and that this helps the body to recover. Other doctors disagree and don't use the medicine, and others use the medicine only when a patient is very unwell (is in "septic shock"). At this site, this project evaluates:

No corticosteroids
A fixed duration of treatment with hydrocortisone for 7 days
Hydrocortisone given only when the patient is in "septic shock"

What are the possible risks and disadvantages of taking part?

Hydrocortisone is a medication that is commonly provided to patients with acute lung infection, and the risks from side effects will be similar whether you participate in the study or not. Your doctor will know what treatment you are receiving at all times, and will be looking out for side effects, and your doctor will also have to agree to you participating in this study. As these are all commonly used drugs, the doctors and nurses who will be caring for you in the ICU are trained to recognize the risks associated with medications. If side effects occur, and the doctor thinks it is best to stop that treatment that is what will happen.

The doctors in this ICU don't know which treatment is best but believe all options are safe and reasonable. Therefore, the choice of whether to use hydrocortisone or not is comparing different types of "standard care".

What are the reproductive risks?

If you could be pregnant or are breast feeding, hydrocortisone is generally safe to use in these situations.

This document is confidential. Please do not share this document with others.

REMAP-CAP
Randomized, Embedded, Multifactorial, Adaptive Platform trial for Community-Acquired Pneumonia

COVID-19 use of statins. Statins have traditionally been used to lower serum cholesterol. They also have anti-inflammatory and antithrombotic (anti-clot) properties which may help prevent and treat patients with COVID-19. Statins may have an effect on viral transmission and infectivity and may potentially decrease the impact of injury to the heart muscles and clotting events associated with severe COVID-19 infection. This project evaluates:

No simvastatin
Simvastatin 80 mg daily up to 28 days

Simvastatin will be given at a dose of 80 mg once-daily through the mouth or feeding tube once daily until ICU discharge or study day 28, whichever occurs first.

The use of statins in COVID-19 is considered investigational because the drugs have not been approved by Health Canada for this purpose. Health Canada has reviewed and authorized their use in this study.

The use of statins is not a usual treatment for COVID-19, so this evaluation is a “new treatment”.

What are the possible risks and disadvantages of taking part?

Side effects of Statins
Simvastatin occasionally causes:

- Myopathy (muscle disease with aching or weakness) which presents as muscle pain (4%).
- Rhabdomyolysis (breakdown of muscle fibers) which is dose-related, especially with the 80 mg dose (1.0%)
- Abnormal kidney function because of rhabdomyolysis – rare
- Liver dysfunction – 1%
- Gastrointestinal disorders – 0.5%
- Upper respiratory infections – 9%
- Headache – 7.4%
- Abdominal pain – 7.3%
- Constipation – 6.6%
- Nausea – 5.4%
- Skin rash – (0.7%)
- Erectile dysfunction - rare

Blood tests which are done as part of your usual care will be monitored for abnormalities that indicate the presence of side effects and this will guide your doctor whether it is best to stop the drug. The side effects usually go away with discontinuation of the drug.

What are the reproductive risks?

There are rare reports of congenital anomalies in infants exposed to statins while in utero. Statins may cause harm to the fetus when taken by a pregnant women. The study doctor will discuss family planning with you to ensure that you do not become pregnant or father a baby during the study.

If you are of childbearing potential, we will request for a pregnancy test prior to enrolment.

This is the end of the prework.

Please save your notes in a safe place so that you can share them at the workshop.

We look forward to seeing you at the workshop!

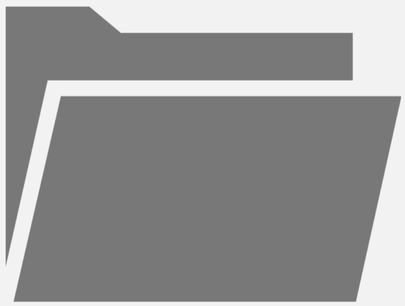

Supplement: Supplementary file 3 — Additional file 3. [file 40814_2023_1290_MOESM3_ESM.pdf]
