# Supplementary material for: Co-designing and pilot testing an infographic to support patients/families through the REMAP-CAP consent process: a mixed-methods study protocol
Source: Pilot Feasibility Stud. 2023 Apr 13;9:58. doi: 10.1186/s40814-023-01290-6 (PMC10098229; doi:10.1186/s40814-023-01290-6)
Supplement: Supplementary file 4 — Additional file 4. [file 40814_2023_1290_MOESM4_ESM.docx]

**Appendix 5:** Semi-structured facilitation guide for phase one focus groups.

**Title:** Pilot Testing an Infographic to Support Patients/Families through the REMAP-CAP Consent Process

**Principal Investigators:** Dr. John Marshall, Dr. Michelle Kho and Heather O’Grady, on behalf of the REMAP-CAP Coordinating Team and the CAPTIC Patient/Family Partners.

**Preamble (Patient/SDM Version):** Thank you for agreeing to participate in our research study to support the REMAP-CAP consent process. As a part of this research study, you will be participating in a focus group discussion. The focus group will consist of 4-6 patients/substitute decision makers and research coordinators and will be approximately 2 hours in length. The purpose of this focus groups is to get feedback on a few prototypes of a resource, consisting of pictures and words, intended to support the consent process. This focus group is completely voluntary - if you are not interested in participating, you may decline.

We understand that participation in this focus group will involve discussing sensitive topics and may re-ignite or worsen feelings of stress, anxiety and/or grief, which are typical of an ICU encounter. As participants, though your full commitment is highly encouraged, you will not have to answer all questions and you may also skip questions which you feel you do not want to answer. You may also pause or leave or exit the focus group at any time.

Lastly, though the focus group discussions will be audio-recorded, all information shared during the workshops will be kept confidential. We also ask that you keep what is shared during the workshop confidential; however, we cannot guarantee that other participants will not share your information or responses. Direct quotes from your responses during the workshop may be used in reports or publications, but the quotes will not be attributed to you or contain any information that could be used to identify you. Your consent to participate will be implied by participating in the workshops. The data collected through these workshops will be kept for a period of at least 7 years in a secure location and then destroyed. You may choose to withdraw your focus group responses at any time by contacting the research study investigators. Your responses will be deleted from the database.

Thank you very much for your participation and we look forward to having you join our research study.

The CAPTIC Research Team

(Include PI Names)

**Preamble (RC Version):** Thank you for agreeing to participate in our research study to support the REMAP-CAP consent process. As a part of this research study, you will be participating in a focus group discussion. The focus group will consist of 4-6 RCs and Patients/SDMs and will be approximately 2 hours in length. The purpose of this focus groups is to get feedback on a few prototypes of a resource, consisting of pictures and words, intended to support the consent process. This focus group is completely voluntary - if you are not interested in participating, you may decline.

As participants, though your full commitment is highly encouraged, you will not have to answer all questions and you may also skip questions which you feel you do not want to answer. You may also pause or leave or exit the focus group at any time.

Lastly, though the focus group discussions will be audio-recorded, all information shared during the workshops will be kept confidential. We also ask that you keep what is shared during the workshop confidential; however, we cannot guarantee that other participants will not share your information or responses. Direct quotes from your responses during the workshop may be used in reports or publications, but the quotes will not be attributed to you or contain any information that could be used to identify you. Your consent to participate will be implied by participating in the workshops. The data collected through these workshops will be kept for a period of at least 7 years in a secure location and then destroyed. You may choose to withdraw your focus group responses at any time by contacting the research study investigators. Your responses will be deleted from the database.

Thank you very much for your participation and we look forward to having you join our research study.

The CAPTIC Research Team

(Include PI Names)

**Activity 1: Likes and Dislikes example questions**

**Prototypes 1-3**

**LIKES**

- What did you like in this prototype? Why?
  - Did anyone else feel the same way?
  - Did anyone feel differently about this? Why?
- What else did you like that hasn’t been mentioned? Why?
  - Did anyone else feel the same way?
  - Did anyone feel differently about this? Why?

**DISLIKES**

- What did you dislike in this prototype? Why?
  - Did anyone else feel the same way?
  - Did anyone feel differently about this? Why?
- What else did you dislike that hasn’t been mentioned? Why?
  - Did anyone else feel the same way?
  - Did anyone feel differently about this? Why?

**GENERAL**

- What overall feeling did you get from this prototype? Why?
  - When you first look at it, did it make you want or not want to read it? Why?
  - Do you feel that it was made for you in mind? Or do you feel that it talks down at you?
  - How would you feel if you had received this while you were in the ICU? Why?
- How would you describe the tone?
  - Do you feel that it’s appropriate for an ICU setting?
- Overall, was it easy or hard for you to understand?

**Activity 2: Refining Content example questions**

**Resource title**

You would be getting this resource when a RC approaches you (as a patient or SDM) to invite you to join REMAP-CAP. The resource is meant to help you understand what you are being invited to and make it easier for you to make your decision.

- Knowing this, what do you think the title of this resource should be?
- Would any of these 3 titles work? Why or why not?
- What would you prefer to call it that would make the most sense to you? Why?

Since REMAP-CAP is type of trial (called a platform trial) and researchers are using this type of trial for this research study, we could potentially call it a ‘study’ or a ‘trial’?

- Does it make more sense to you to call it a ‘study’ or a ‘trial’? Why?
  - What does ‘(research) study’ mean to you?
  - What does ‘trial’ mean to you?
- Is there another way of calling REMAP-CAP that would be more meaningful to you? Why?

**REMAP-CAP definition**

REMAP-CAP stands for Randomized, Embedded, Multi-factorial, Adaptive Platform Trial for Community-Acquired Pneumonia.

- Do you feel that it’s enough to know how the trial works and how you would be involved?
- Or is it also important to you that you know what REMAP-CAP stands for? Why?

Based on the definition, we know REMAP-CAP is a specific type of trial called a platform trial. Prototype 1 specifies that REMAP-CAP is a platform trial. In Prototypes 2 and 3, however, REMAP-CAP is referred to as simply a ‘trial’ or a ‘study’ alongside a section on how this ‘trial’ or ‘study’ works.

- How do you feel about the term ‘platform trial’? Is it important to you that the resource specifies that REMAP-CAP is a ‘platform trial’ and not just ‘trial’ or ‘study’? Why or why not?

**Contact info**

On the top right of Prototype 2, there is a box for the RC to write down their name, their contact info, and a time to check back in with you if you choose to take a bit more time to make your decision.

**From a patient perspective:**

- Is this a feature that you would find useful? Why?
- How do you feel about the wording used for the three items?
- Is it clear to you what each item means?
- If not, what wording do you feel is clearer to you?

**From a RC perspective:**

- Would you use this?
- How do you feel about the wording used for the three items?
- Is it clear to you what each item means?
- If not, what wording do you feel is clearer to you?

**Participation**

When we talk about participation in the trial, we can talk about how it’s completely voluntary and how joining the trial may impact your existing care.

- Are both points something that is important for you to know? Why or why not?
- What other questions around participation would you have?

**VOLUNTARY**

- When communicating that the trial is voluntary, which wording do you prefer between Prototypes 2 and 3? Why?
- How does the wording in each make you feel? What tone does it have?
- Is the message clear? Could it be confusing for others?
- Does the wording make you want to or not want to join the trial? Why?
- How would you like to see this worded?

**IMPACT**

- When communicating how joining the trial may or may not impact your existing care, which wording do you prefer between Prototypes 2 and 3? Why?
- How does the wording in each make you feel?
- Is the message clear? Could it be confusing for others?
- Does the wording make you want to or not want to join the trial? Why?
- How would you like to see this worded?

**Benefits and risks**

**BENEFITS**

There is currently no definite direct benefits to you to joining the trial. Prototype 2 focuses on what we do know, which is the benefit to future patients. In Prototype 3, it directly addresses that there may or may not be direct benefits to you but also mentions that there’s benefits to others.

- Which approach do you prefer between Prototypes 2 and 3? Why?
- Is it important for you to know that there are no definite direct benefits to you? Why?
- Or is it enough to focus on where there is a benefit, which is benefit to future patients?
- How does the wording make you feel? What tone does it have?
- Is it clear? Could it be confusing for other people you know?
- Does the wording make you want to or not want to join the trial? Why?
- How would you like to see this worded?
- How might we make it short and clear without discouraging people from joining the trial?

**RISKS**

When communicating the risks, Prototype 2 doesn’t directly mention risk, but mentions that the RC will go over everything with you and you can ask questions (about the risks). On the other hand, Prototype 3 directly addresses risk.

- Which approach of communicating risks do you prefer?
- How does the wording make you feel?
- Is it clear? Could it be confusing for other people you know?
- Does the wording make you want to or not want to join the trial? Why?
- How would you like to see this worded?
- Do you have other questions around benefits and risks that is not addressed here?

**REMAP-CAP vs traditional trials**

As a platform trial, REMAP-CAP is a bit more complex than how many people think a medical trial works.

**TRADITIONAL TRIALS**

- Is it important for you to know how REMAP-CAP is different from a ‘traditional’ trial?
- What does “traditional’ trial mean to you? How does it work?
- What do you feel would be a better term to use instead of ‘traditional’ trial? Why?

**DIFFERENCES**

- When communicating the differences, which wording do you prefer between Prototypes 1-3? Why?
- Is the difference clear to you? Could it be confusing for others? (e.g. your parents)
- How might we make this easier to understand?

**VISUALS**

- How do you feel about the visuals used in Prototype 3?
- Do the visuals make it easier or harder to understand? Why?
- How might we tweak these visuals or use new visuals to make this easier to understand?

**REMAP-CAP vs standard care**

We’ve heard from some patients that they want to know if what they get in the trial is different from what they would get if they’re not in the trial.

- Is this something that is important for you to know before you make your decision? Why?

**DIFFERENCES**

On the back of Prototype 2, under each domain, it specifies whether the interventions in that domain are part of standard care. But in Prototype 3, there is a side-by-side explanation of standard care versus REMAP-CAP.

- Which explanation tells you what you want to know? Why?

For the explanation in Prototype 3:

- Is this explanation clear to you? If not, why?
- Did any part of it raise more questions for you? Why?
- Is it important for you to know that even if you don’t join the trial, you might still get some of the same interventions that are being tested in the trial as part of your care?
- Does this explanation affect whether you would want to join the trial? Why?

**DATA COLLECTION**

Another difference that is not mentioned in either prototypes is the fact that if you’re on the trial, researchers will collect data on how you’re doing on an intervention. But if you’re not in the trial, and you are getting the same intervention as part of standard care, there will be no data collected.

- Is it important for you to know that your health data is being collected in the trial? Why?
- Does knowing that your data is collected affect whether you would want to join the trial? Why?

**How REMAP-CAP works**

**ANALOGY**

Prototypes 2 and 3 use different analogies to explain how REMAP-CAP works. Prototype 2 uses a menu analogy and 3 uses a playing card analogy.

- Which analogy was easier for you to understand? Why?
- Which was more relatable to you? Why?
- Is there any cultural reasons to why you would prefer one over the other? For example, card playing may be negatively associated with gambling in some cultures.
- Was there anything that confused you in either analogies or raised more questions for you? Which parts and why?

**IMAGERY**

Prototype 2 uses images of a simplified version of the menu on the back of the page and cartoon style people.

- Did these images make it easier for you to understand or did they make it worse for you? Why?
- Did the images make you want to read it it? Why?
- Do you feel that the images are appropriate for an ICU setting?
- Do you feel that the people shown should be accurate to an ICU setting? (e.g. laying in a bed)
- How do you feel about the choice of people depicted? (in terms of gender, ethnicity, diversity)

Prototype 3 uses colourful images of playing cards.

- Did these images make it easier for you to understand or did it confuse you at any point? Why?
- Did the images make you want to read it it? Why?
- Is there a different way you would have preferred the cards to be shown? Why?

**RANDOMIZATION**

Prototype 2 tells you that once you’ve finished editing your menu with your RC, your healthcare team will choose one or more domains from your menu and a computer will then choose interventions from these domains for you.

- How do you feel about whether it is a person choosing or a computer choosing? Why?

In reality, as the computer continues to collect and analyze data from the trial, it will know which interventions seem to be doing better. When a new patient joins the trial and the computer is choosing which interventions to give the patient, the computer is more likely to choose the interventions that have been doing better. This point is implied by the bubble and the computer image.

- Was this how you understood the message in the bubble? If not, what did you think it meant?
- Is this something that you feel is important for you to know? Why?
- Does knowing this affect whether you want to join the trial? Why?
- How might we make this point clearer?

Prototype 3 doesn’t mention that there’s a higher chance for the computer to choose interventions that have worked better for others.

- Is this something that is important to add in? Why?

**TRIAL OUTCOMES**

The ’how the trial works’ parts of Prototypes 2 and 3 are only part of the bigger research timeline. It covers the point from when the RC is first preparing the menu/cards to invite you to the trial to when you decide to join the trial and receive the selected interventions.

- Is it important to you to know what researchers have learned in the trial before approaching you for the first time? Why?
- Is it important to you to know what they are learning in the trial as you are going through it? Why?
- After you have left or completed the trial yourself, is it important to you to be updated on what they are learning in the trial? Why?

In Prototype 1, it specifically mentions that interventions are continuously analyzed during the trial, and that new patients will be more likely to be given better performing domains.

- Do you feel that any of these points are important to include in the resource? Which points and why?
- Does the point affect whether you would want to join the trial? Why or why not?

**Domains**

Prototype 1 provides only the name of the domain. Prototype 3 gives a short sentence description about the domain (e.g. what the meds do), and Prototype 2 gives the longest description about the domain (e.g. what the meds do and how it may help for COVID).

- Which pieces of information do you feel is important for you to know about a domain? Why?

If the resource included everything you needed to know about the domain, it would be a lot of text on the page (similar to Prototype 2).

- In order to cut down the text on the page, what pieces of information would you choose to cut out? Why?

**Interventions**

When listing the individual interventions within a domain, it can be just the name of a drug (e.g. azithromycin) or a longer and more complicated description (e.g. Continuation of therapeutic dose anticoagulation).

- Is it important for you to know the specific names of interventions? Why?
- Would you prefer the proper name of the intervention, or would you prefer a simplified version of it when possible? Why?
- Would you want to know which specific interventions you end up getting in the trial? Why?

Prototypes 1 and 3 only provide the name of the intervention, whereas Prototype 2 goes into a lot more detail (e.g. whether the interventions are part of standard care, the intervention options, the research question, and how you get the intervention).

- Which pieces of information do you feel is important for you to know about an intervention so that you can decide whether you feel comfortable with getting it? Why?
- Which pieces of information do you feel need to be in this resource versus just being in the consent form or verbally explained by the RC? Why?
- Is it important for you to know whether a specific intervention is part of standard care? Or do you feel that it’s enough for you to know on a high level that some interventions in the trial are also part of standard care? Why?
- Would information about how you get an intervention (e.g. feeding tube or injection) affects whether you would join the trial? Why?
- Is the space for notes something you would use? Why?
- If we wanted to cut down the amount of text on the page, which pieces of information would you choose to cut first? Why?

**Usability**

Let’s quickly review how Prototypes 2 and 3 are meant to be used:

1. All the domains and interventions shown are meant to reflect what is currently being tested in the trial at a specific hospital.
2. Before meeting the patient, RCs need to cross out domains that don’t work for the patient based on their condition.
3. During the meeting with the patient, the patient might decide together with the RC to cross a few more domains off if they are uncomfortable with anything.
4. The patient makes their decision on whether to join the trial.
5. If they decide to join, the RC will come back to the patient and check off the interventions that the patient will be receiving in the trial.

**From a patient perspective:**

- Is it important to you to see the full list of domains/interventions (e.g. 6 domains) that is being tested even though when you get the resource, you may see that some domains have been crossed out? Why?
- Or do you feel that you would rather only see the domains that your RC thinks are most suitable for you (e.g. 4 domains)? Why?
  - If so, is it important to you to know that you’re not seeing the full list and that the RCs has removed some options?
  - Should this part of the process be removed from the “how the trial works” section?

**From a RC perspective:**

This resource is built in PowerPoint so that it can be edited and updated as needed.

- Do you know how to use PowerPoint or is there another program you are more comfortable with?
- Does the prep work (i.e. crossing out domains) work with your workflow or your site’s existing processes? Why or why not?
- Do you currently go back to the patient to tell them what interventions have been chosen for them?
- Do you think you can/would go back to check off the interventions for the patient?
- How might this resource be tweaked to make it easier for you to edit or better fit into existing processes?

**Link to additional resources**

We know that you’ll be receiving a lot of information about the trial when the RC invites you to join it: you will be receiving this resource, the consent form, and the RC will be verbally going through the materials with you before you make your decision.

- Between all this, do you feel that you have what you need to make your decision? Why?
- If not, what do you feel is missing? Why?
- What would prevent you from making a decision?
- Where would you turn to to get the information you need in order to make your decision? Why?

In Prototype 1, there are two additional resources shared: a QR code to a video on how the trial works and a link to the REMAP-CAP international research website.

**VIDEO**

- Did you use the QR code to view the video? Why or why not?
- If you watched the video, what did you think of it? Why?
- Did the video give you any helpful information that’s not in the resource or the consent form? What did you learn?

**REMAP-CAP WEBSITE**

- Did you go to the website? Why or why not?
- If you went to the website, what did you think of it? Why?
- Did the website give you any helpful information that’s not in the resource or the consent form? What did you learn?

**Glossary of terms**

In Prototypes 2 and 3, when a new terminology is introduced (e.g. intervention), it’s immediately followed by an explanation of what it means or instead of using the terminology, the resource will use simpler words in place of it. But in Prototype 1, there is a glossary of terms on the back to explain all the terminology used in the resource.

- Which approach do you prefer when learning new medical terms? Why?
- Do you prefer to learn and use the medical terms your healthcare team uses (e.g. anti-coagulants) or have your healthcare team communicate with you in terms you are more familiar with (e.g. blood thinners)? Why?
- Do the different approaches impact your willingness to join the trial? Why?
- Do the different approaches impact how you feel about your healthcare team? Why?
